# Synthesis of natural 1-*O*-alkylglycerols: a study on the chemoselective opening of the epoxide ring by onium quaternary salts (N and P) and ionic liquids†

**DOI:** 10.1039/c9ra09217j

**Published:** 2020-01-03

**Authors:** Thiana Santiago Nascimento, Esther Faria Braga, Giselle Cristina Casaes Gomes, William Romão Batista, André Luís Mazzei Albert, Rosangela Sabbatini Capella Lopes, Claudio Cerqueira Lopes

**Affiliations:** Laboratório de Síntese e Análise de Produtos Estratégicos-Centro de Tecnologia, Departamento de Química Analítica, Instituto de Química, Universidade Federal do Rio de Janeiro Bloco A, S. 508 Rio de Janeiro-RJ CEP 21941-909 Brazil claudiosabbatini@uol.com.br

## Abstract

A chemoselective route for the synthesis of 1-*O*-alkylglycerols chimyl (1), batyl (2), and selachyl (3) is reported. These compounds can be naturally isolated from shark liver oil and the skin of animals such as stingrays and chimeras and exhibit potential anti-fouling activity. The synthetic approach developed in this work included two distinct methods of preparation. The first was based on solvent-free reactions catalyzed by onium quaternary salts (N and P) and ionic liquids; the second methodology was based on a series of one-pot reactions.

## Abstract 2

A chemoselective route for the synthesis of 1-*O*-alkylglycerols chimyl (1), batyl (2), and selachyl (3) is reported.

## Graphical abstract

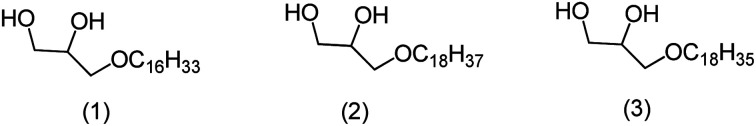

## Introduction

Natural 1-*O*-alkylglycerols (alkyl-Gro) are bioactive lipid ethers present in cells and body fluids of marine animals. They are the precursors of phospholipid ethers which participate in the structure and membrane functions of certain cells such as red blood corpuscles and macrophages.^1,2^

Regarding marine sources, 1-*O*-alkylglycerols (1, 2, 3) may be found in shark liver oil and the skin of stingrays and chimeras (marine animals of the *Chimaeridae*, *Bathoidea* and *Selachoidea* families).^3^ These organisms possess high levels of the aforementioned compounds with a mixture of various chain lengths, abundancy, and position of the unsaturation.^4–7^ For example, the composition of alkyl chains in 1-*O*-alkylglycerols in the shark liver oil of *Centrophorus squamosus*, also known as Leafscale gulper shark, has the following values: 12 : 0, 1–2%; 14 : 0, 1–3%; 16 : 0, 9–13%; 16 : 1n-7, 11–13%; 18 : 0, 1–5%; 18 : 1n-9, 54–68%; 18 : 1n-7, 4–6%.^4–6^

**Scheme 1:**
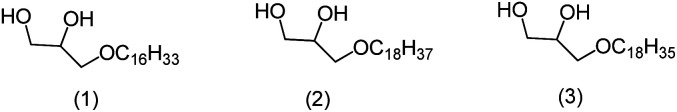
Representative structures of 1-*O*-alkylglycerols.

The 1-*O*-alkylglycerols in the shark liver oil present the ether moiety with the following profile: C16:0, chimyl alcohol (1); C18:0, batyl alcohol (2); C18:1, selachyl alcohol (3) (Scheme 1). The latter being in greater amount.^8^

Some of the beneficial effects related to 1-*O*-alkylglycerols have already been described in the literature. Among them is the ability to promote important stimulation for the immunological control, production of antibodies, and anti-inflammatory effects.^9,10^

An investigation in the field of medicine on patients diagnosed with cervical cancer showed great recession on the size of the tumor when submitted to radiation treatment alongside the administration of mixtures of natural 1-*O*-alkylglycerols 1, 2, 3.^11–13^

Furthermore, recent studies have been suggesting the use of these 1-*O*-alkylglycerols 1, 2, and 3 as anti-fouling agents and evaluating their efficiency towards fouling processes on metallic surfaces of ships, platforms, and oil pipelines.^13^

The methodologies developed with the goal of evaluating the biological activity of 1-*O*-alkylglycerols suggest that they may be incorporated in phospholipids of cell membrane leading to changes in its physical properties, *e.g.* fluidity, or alter cell signaling.^14–16^

There are reports of some preparation methods of 1-*O*-alkylglycerols in the literature using derivatives of glycerol as starting materials.^13,17–25^

Some studies mention the synthesis of compounds 1, 2, and 3 from solketal, the isopropylidene ketal of glycerol.^20–22^ These 1-*O*-alkylglycerols can also be synthetically obtained from glycidol, an epoxide of chemical structure similar to that of epichlorohydrin with a hydroxyl group.^23^

Within the perspective of waste recovery from the industries of refined soy oil and biodiesel produced in Brazil, we used, in a recent study, starting materials such as epichlorohydrin^13^ and soy lecitins^24^ for the synthesis of potential biocides. We also demonstrated that lyso-glycerophosphocholines prepared from soy lecithins display potential biocide activity on the treatment of ballast water.^24^

Herein, we prepared 1-*O*-alkylglycerols 1, 2, and 3 found in the skin and liver of sharks, stingrays, and chimeras, marine animals that do not present biofouling. This characteristic suggests that these substances (1, 2, and 3) are potential candidates to be incorporated in anti-fouling paints as biocides, becoming a green chemistry option to control the process of biofouling that leads to enormous losses for sea transport.^13^

It is possible to notice that epichlorohydrin, an organochlorine compound used in various industrial applications, has been gaining relevance in the synthesis of 1-*O*-alkylglycerols.^13,23,24^ Epichlorohydrin is obtained from glycerol, which is itself a by-product of the biodiesel production (biomass fuel) and was used in this study as the starting material.

In most commercial marine paint coatings the copper ions are incorporated as biocide to combat marine biofouling. The previous biological results demonstrated by our research group^13^ indicated that chimyl (1) can be used as a promising biocide because it is more effective than copper ions against biofilm-forming bacterias involved on marine biofouling process.

We are making, on macro scale, new marine paints including the natural 1-*O*-alkylglycerols chimyl (1), batyl (2), and selachyl (3) as biocide additive, on different concentrations as a new technological contribution to prevent the formation of biofilm related with the marine biofouling process. All these compounds were prepared with the synthetic route described herein.

The aim of this study was to develop a methodology with a reduced number of steps, ease of execution, and high yields, in order to allow for the large-scale synthesis of compounds 1, 2 and 3.

## Results and discussion

**Scheme 2:**
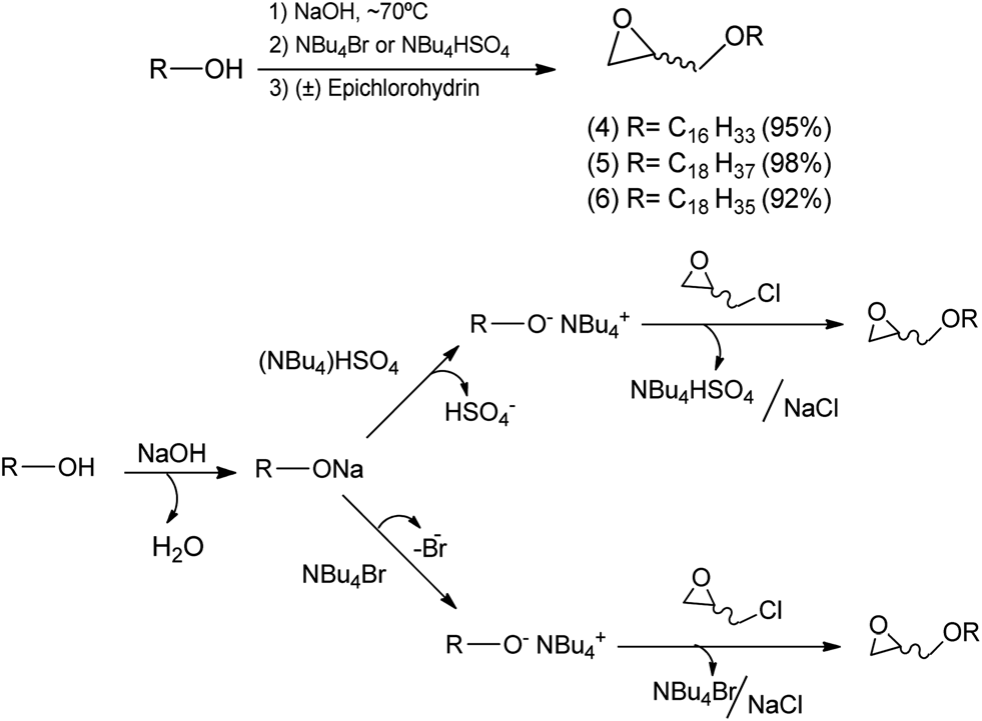
Mechanistic proposal for the formation of glycidyl ethers 4, 5 and 6.

A reaction medium containing an alcohol of long aliphatic chain, such as cetyl, stearyl, and oleyl alcohols, and epichlorohydrin in the presence of a quaternary ammonium salt phase-transfer catalyst, in alkaline and solvent-free medium was kept under stirring at 70 °C for 7 hours. This reaction led to the formation of glycidyl ethers 4, 5, and 6 with yields from 92–98% (Scheme 2). In the formation of the glycidyl ethers, two different ammonium quaternary salts were used, tetrabutylammonium bromide (TBAB) and tetrabutylammonium hydrogen sulfate (TBAHS), with the latter providing better yields for compounds 4, 5, and 6.

Our experimental data show that the HSO_4_^−^ ion is a slightly better leaving group than the bromide ion. Therefore, we obtained better yields when TBAHS was used as catalyst instead of TBAB. A mechanistic proposal for the formation of glycidyl ethers 4, 5 and 6 is described on Scheme 2. It was possible to notice from this reaction that high yields are obtained with a solvent-free system, a phase-transfer catalyst with acid characteristics (such as TBAHS), and moderate temperatures. A similar methodology was used by Kang^25^ and coworkers in a study that involved the use of phase-transfer catalysts in the synthesis of similar glycidyl ethers.

The second step of the synthetic route for the preparation of 1-*O*-alkylglycerols 1, 2, and 3 consisted of an epoxide ring-opening reaction using benzoic acid and involving the improvement of the reaction conditions, such as reaction time, catalyst concentration, and presence or absence of the solvent, in the search for reproducible yields.

Previously, the epoxide ring opening reaction (second step) had been performed under different temperature conditions, where the temperature was increased 20 °C at a time ranging from 30° to 110 °C. This screening showed that the reaction had best performance at 110 °C using toluene as the solvent.

**Scheme 3:**
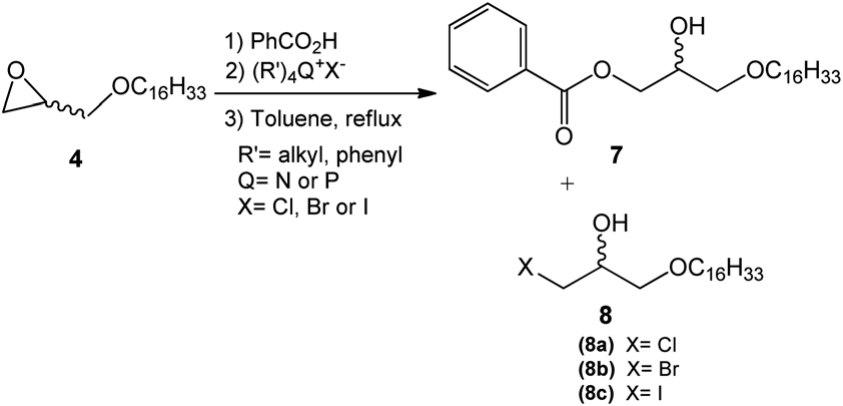
Epoxide ring-opening mediated by an onium quaternary salt (N, P).

This step consisted of keeping a mixture of glycidyl ether 4 with benzoic acid in the presence of an onium salt (N or P) in toluene and under reflux (Scheme 3).

Fifteen commercial quaternary salts containing a nitrogen or phosphorus central atom with different R groups and halides (–Cl, –Br, –I) were used in the ring-opening of compound 4 (Scheme 3).

The compilation of the results presented in Table 1 showed that a variety of quaternary ammonium and phosphonium salts adequately assist the epoxide ring-opening process, favoring the hydroxy ester intermediate 7.

**Table 1:** Epoxide ring opening of glycidyl ether 4 catalyzed by quaternary ammonium or phosphonium salt^a^^,^^b^

Entry	Surfactant	(7)^b^ %	(8a, 8b or 8c)^b^ %	Reaction time
1	C_16_H_33_N Me_3_Br	80	20	20 h
2	C_16_H_33_N Me_3_Cl	80	20	20 h
3	*n*-Bu_4_NI	90	10	14 h
4	*n*-Bu_4_NBr	80	20	13 h
5	*n*-Bu_4_PBr	80	20	9 h
6	MePPh_3_Br	90	10	9 h
7	PropPPh_3_Br	80	20	13 h
8	BuPPh_3_Br	80	20	15 h
9	PentPPh_3_Br	80	20	14 h
10	HexPPh_3_Br	80	20	15 h
11	MePPh_3_I	95	∼5	7 h
12	PropPPh_3_I	80	20	9 h
13	BuPPh_3_I	70	30	9 h
14	PentPPh_3_I	70	30	12 h
15	HexPPh_3_I	70	30	10 h

a Reaction conditions: 4 (1 mmol), PhCO_2_H (2 mmol), surfactant [(R′)_4_QX] (25 mmol%), reflux (*T* = 100 °C).

b Isolated yield after flash chromatography.

Furthermore, the most efficient surfactant among the analyzed salts was methyltriphenylphosphonium iodide (entry 11), producing intermediate 7 with a shorter reaction time (7 h) and 95% yield.

According to the data presented, it is possible to infer that the process is highly regioselective given that the formation of the monohydroxy ester 7 is preferred in most reactions. The yields of the formation of halohydrin 8a, 8b, and 8c (X = Cl, Br, I respectively) ranged from 5 to 30%. Halohydrins 8a, 8b, and 8c are generated when the halide ion provided by the quaternary salt attacks the least substituted carbon in the epoxide ring leading to its opening.

**Scheme 4:**
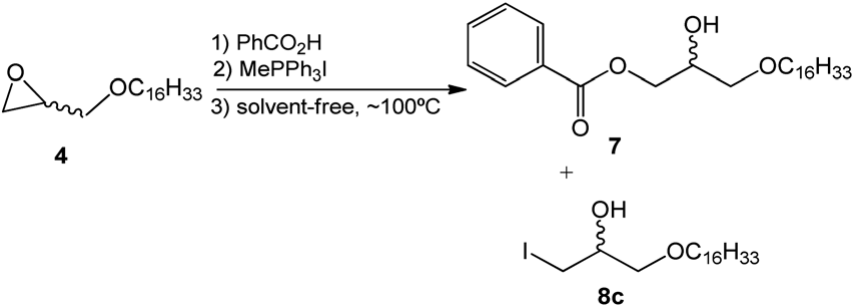
Epoxide ring-opening of 4 catalyzed by methyltriphenylphosphonium without solvent.

Considering the results obtained and reaction factors such as solvent effect and surfactant molar concentration, a solvent-free ring-opening reaction was developed using the methyltriphenylphosphonium salt (Scheme 4).

In this synthetic approach to the opening of the epoxide ring the concentration of the reagents, which was initially optimized in the wet-method, was maintained. This strategy allowed the conclusion that the solvent-free reaction leads to similar results when compared to the reaction in toluene. However, the time needed for the completion of the reaction (4.0 hours) and the amount of collateral product 8c (X = I) are both reduced (Table 2).

**Table 2:** Solvent-free opening of the epoxide ring of compound 7 catalyzed by onium quaternary salt^a^^,^^b^

Entry	Surfactant	(7)^b^ %	(8c)^b^ %
1	MePPh_3_I (25 mol%)	∼95	∼5
2	MePPh_3_I (12.5 mol%)	∼95	<5
3	MePPh_3_I (10 mol%)	100	Trace
4	MePPh_3_I (5 mol%)	100	—
5	MePPh_3_I (2.5 mol%)	100	—
6	MePPh_3_I (1 mol%)	100	—

a Reaction conditions: 4 (1 mmol), PhCO_2_H (2 mmol), surfactant (MePPh_3_I) (25 mmol%–1 mmol %), solvent-free reaction. *T* = 100 °C.

b Isolated yield after flash chromatography.

The ideal concentration of the phosphonium quaternary salt was also analyzed. According to the data presented in Table 2, it is possible to infer that there is a correlation between surfactant concentration and the yield of the hydroxy ester 7, since the lower the concentration of the surfactant, the higher the yield of the hydroxy ester. Thereby, a solvent-free system and catalytic amounts of the quaternary salt favor the evolution of the reaction.

**Scheme 5:**
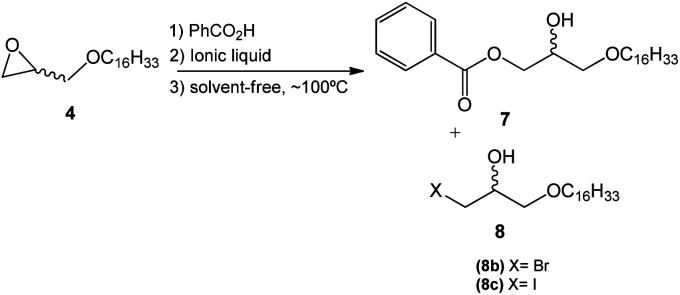
Opening of the epoxide ring in compound 4 using the ionic liquids.

Additionally, the influence of several ionic liquids (ILs) – alternative catalysts for the onium quaternary salts – in the process of opening the epoxide ring in the substrates already used was investigated. The experimental protocol for this study consisted in a water/solvent-free reaction using glycidyl ether 4, in the presence of benzoic acid and the ionic liquid at 110 °C. This particular type of reaction becomes interesting given the fact that ionic liquids allow a homogeneous system without the need of further organic solvents (Scheme 5).

In order to analyze the catalytic efficiency of the ionic liquids in the epoxide ring-opening reaction, compound 4 was used along with eight commercial ionic liquids derived from imidazole. To facilitate the identification of the substances in the course of the discussion, the ionic liquids will be referred to by the first letter of their carbonyl chain and halides, for instance, 1,3-dimethylimidazole iodide will be dubbed [MMIm]I, where the methyl groups will be represented by the letter M, the imidazole group by Im, and the iodide by I. The code for the other ionic liquids will follow the same pattern and logic, as seen in Table 3.

**Table 3:** Epoxide ring-opening using ionic liquids^a^^,^^b^^,^^c^

Entry	Ionic liquid	% (7)^c^	% (8b) or (8c)^c^	Reaction time
1	[MMIm]I	∼100	—	5 h
2	[PMIm]I	∼95	Trace	5 h
3	[BMIm]I	∼95	Trace	7 h
4	[HMIm]I	∼95	Trace	7 h
5	[PMIm]Br	95	∼5	6 h
6	[BMIm]Br	95	∼5	6 h
7	[PentMIm]Br	95	∼5	7 h
8	[HMIm]Br	95	∼5	7 h

a M = methyl; P = propyl; B = butyl; Pent = pentyl; H = hexyl; Im = imidazole; I = iodide; Br = bromide.

b Reaction conditions: 4 (1 mmol), PhCO_2_H (2 mmol), ionic liquid (1 mmol%). *T* = 100 °C.

c Isolated yield after flash chromatography.

The investigation on the use of ILs showed that the promotion of a more homogeneous reaction environment even in solvent-free conditions allows the reagents to interact in a more effective way, enhancing the efficacy of the collision between the molecules and leading to the decrease of reaction time to form compound 7. The results presented in Table 3 indicate that 1,3-dimethylimidazole iodide (entry 1) can be considered the most convenient for the epoxide ring-opening reaction.

After confirming the optimal conditions for the preparation of compound 7 – ideal concentration and type of catalyst – they were extended to substances 9 and 10 (the hydroxyl esters containing stearyl (18:0) and oleyl (18:n9) groups, respectively, in the 1-*O*-alkyl chain).

**Scheme 6:**
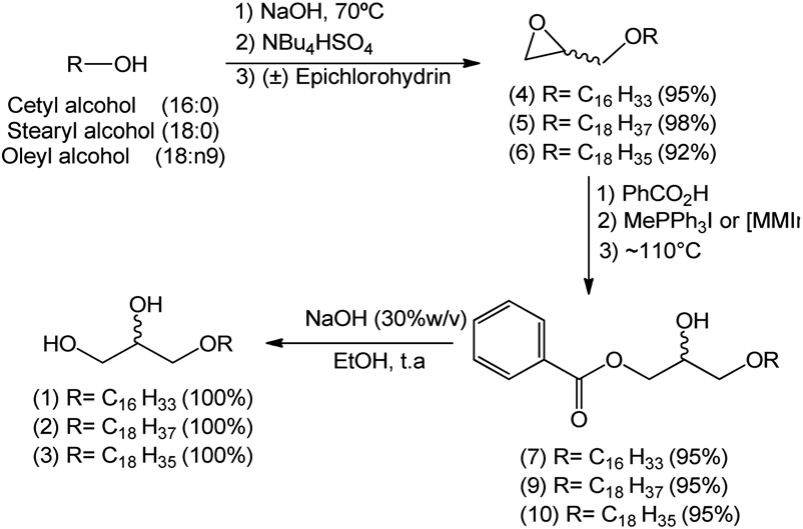
Total synthesis of 1-*O*-alkyl glycerols 1, 2 and 3.

The final step consisted in the treatment of hydroxy esters 7, 9 and 10 with a basic solution (NaOH 30%) to convert them into the natural 1-*O*-alkylglycerols 1, 2 and 3 in quantitative yields (Scheme 6).

After achieving the optimal conditions for all three steps leading to the formation of compounds 1, 2 and 3, an one-pot reaction was used in order to replicate this synthetic strategy in a macromolar scale in the future. The one-pot methodology appears to be ideal since it avoids tedious separation/extraction processes, purification of intermediates, saves time, resources and in some cases leads to better yields.

**Scheme 7:**
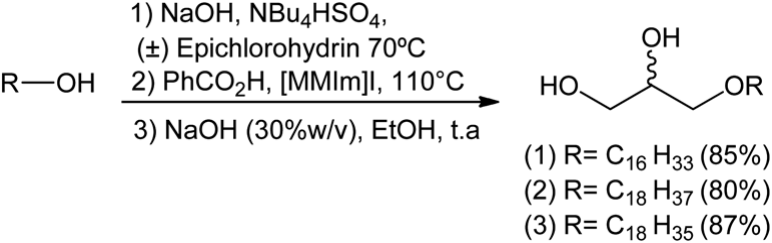
Synthetic strategy for 1-*O*-alkyl glycerols 1, 2 and 3 by one-pot reaction.

In this approach, the sequence of reactions is initiated by the formation of glycidyl ethers 4, 5 and 6. Next, there is the conversion to the corresponding hydroxyl esters 7, 9, and 10 by the action of benzoic acid catalyzed by a surfactant (MePPh_3_I or 1,3-dimethylimidazole). Hydroxy esters 7, 9 and 10 are turned into the aimed 1-*O*-alkyl glycerols 1, 2, and 3 by basic hydrolysis. In the conditions used, 1-*O*-alkylglycerols 1, 2, and 3 were obtained by the one-pot method in yields of 85%, 80% and 87%, respectively. The total synthesis of 1, 2 and 3 is described on Scheme 7.

It was observed that the yields obtained by the one-pot method were lower for compounds 1 (85%) and 2 (80%) when compared to the conventional method (Scheme 7). In the latter, compounds 1, 2, and 3 were obtained in overall yields of 90%, 93%, 87%, respectively. This may be associated with the time required for the maximum conversion of epichlorohydrin to glycidyl ether, and operational errors during purification of the targeted product which is relatively polar.

It is worth of mention that the yields in this work were calculated through the mass/mass ratio of isolated pure products and some were purified through conventional flash chromatography.

## Conclusion

The first step of this study consisted of the synthesis of the three glycidyl ethers 4, 5, and 6 through a solvent-free reaction using epichlorohydrin as starting material in the presence of long chain alcohols – cetyl, stearyl and oleyl, and having tetrabutylammonium hydrogen sulfate as phase-transfer catalyst in basic medium. This methodology proved to be efficient, robust and of easy experimental execution, allowing the preparation of desired compounds 4, 5, and 6 in high yields (95, 98, 92% respectively).

In the second step, the preparation of hydroxyl esters 7, 9, and 10 was developed starting with a chemoselective epoxide ring-opening reaction of glycidyl ethers 4, 5, and 6, employing ammonium/phosphonium salts and ionic liquids. In all cases, the preferential formation of mono-esters 7, 9, and 10 was observed. Moreover, among the ammonium/phosphonium salts studied, methyltriphenylphosphonium iodide was shown to be the most selective. The ILs turned out to be alternative catalysts to the onium quaternary salts, given that they favor the formation of intermediates 7, 9, and 10, decreasing the reaction time in solvent-free conditions.

The third step involved the preparation of 1-*O*-alkylglycerols 1, 2, and 3, which were obtained by a basic hydrolysis of hydroxyl esters 7, 9, and 10 in alcoholic medium with full conversion.

In conclusion, the pathways followed for the preparation of chimyl (1), batyl (2) and selachyl (3) alcohols enabled the combination of these reactions in order to produce compounds 1, 2, and 3 through a one-pot reaction series. This strategy is particularly interesting for the large-scale production of said compounds given that it avoids extractions of the intermediates, diminishes the number of steps, saves time and reagents in addition to generating less residues for the environment.

## Conflicts of interest

The authors declare no competing financial interest.

## Supplementary Material

RA-010-C9RA09217J-s001
