# Supplementary material for: Synthesis of natural 1-O-alkylglycerols: a study on the chemoselective opening of the epoxide ring by onium quaternary salts (N and P) and ionic liquids
Source: RSC Adv. 2020 Jan 3;10(2):1050–4. doi: 10.1039/c9ra09217j (PMC9047497; doi:10.1039/c9ra09217j)
Supplement: RA-010-C9RA09217J-s001 [file RA-010-C9RA09217J-s001.pdf]

Electronic Supplementary Material (ESI) for RSC Advances.
This journal is © The Royal Society of Chemistry 2019

SUPPORTING INFORMATION

Synthesis of natural 1-O- alkylglycerols: A study on the chemoselective opening of the epoxide ring by onium quaternary salts (N and P) and ionic liquids.

Thiana Santiago Nascimento, Esther Faria Braga, Giselle Cristina Casaes Gomes, William Romão Batista, André Luís Mazzei Albert, Rosangela Sabbatini Capella Lopes, Claudio Cerqueira Lopes *

Universidade Federal do Rio de Janeiro, Instituto de Química, Departamento de Química Analítica, Laboratório de Síntese e Análise de Produtos Estratégicos- Centro de Tecnologia, Bloco A, S. 508, Rio de Janeiro-RJ, CEP 21941-909, Brazil.

* Email address: **claudiosabbatini@uol.com.br**

Table of Contents

1. General Information 3
2. Experimental Section..3
3. The 1D NMR spectra of 1-10..4
4. ESI-HRMS analysis for the 7-10..6
5. Copies of 1D NMR spectra of 1-10..9
6. The 2D NMR (COSY) of 7, 9 and 10 25

1. General Information

Unless otherwise noted, all reagents and solvents were obtained commercially and used without further purification. Column chromatography on silica gel (300-400 mesh) was carried out using technical grade and analytical grade (without further purification). ^{1}H and ^{13}C spectra were recorded on a 300 MHz, 400 MHz or 500 MHz spectrometer. Chemical shifts (δ) were reported in parts per million (ppm) downfield from tetramethylsilane (TMS δ = 0.00). ^{1}H NMR spectra were referenced to $CDCl_3$ (7.27 ppm) and ^{13}C NMR spectra were referenced to $CDCl_3$ (77.0 ppm). Peak multiplicities were designated by the following abbreviations: s, singlet; d, doublet; t, triplet; m, multiplet; brs, broad singlet and J, coupling constant in Hz. HRMS spectra were recorded with Micromass QTOF2 Quadrupole/Time-of-Flight Tandem mass spectrometer using electron spray ionization.

2. Experimental Section

2.1 Synthesis of 1-O-alkylglycerols 1-3

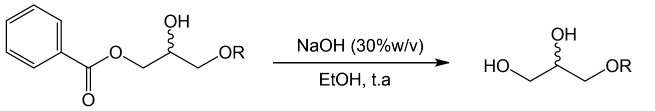

(7) R= $C_{16}H_{33}$ (95%)
(9) R= $C_{18}H_{37}$ (95%)
(10) R= $C_{18}H_{35}$ (95%)

(1) R= $C_{16}H_{33}$ (100%)
(2) R= $C_{18}H_{37}$ (100%)
(3) R= $C_{18}H_{35}$ (100%)

The hydroxy ester was added to a solution of sodium hydroxide (2.5 mmol: 30% sodium hydroxide) and ethanol (50 mL). The reaction medium was kept under stirring at room temperature for 10 hours. The product was extracted with ethyl acetate (50 mL) and washed with distilled water (6x10 mL). Next, the organic solvent was evaporated in a vacuum pump to achieve 1-*O*-alkylglycerols.

2.2 Synthesis of glycidyl ethers 4-6

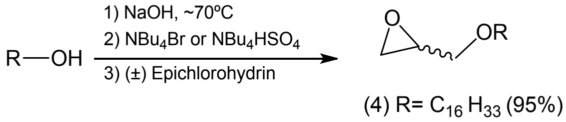

(4) R= $C_{16}H_{33}$ (95%)
(5) R= $C_{18}H_{37}$ (98%)
(6) R= $C_{18}H_{35}$ (92%)

The alcohols of long aliphatic chain (1.0 mmol) were melted in a round bottom flask in the presence of tetrabutylammonium bromide (TBAB) or tetrabutylammonium hydrogen sulfate (TBAHS) (6.25×10^{-3} mmol) at 55ºC. The temperature was then elevated to 70°C, sodium hydroxide (1.5 mmol) was added, and the mixture was stirred for 20 minutes. Epichlorohydrin (2.0 mmol) was added to the medium under vigorous stirring. The reaction was monitored by thin layer cromatography (TLC). The reaction mixture was quenched when the amount of product formed was kept constant. The product formed was extracted with n-hexane, washed with brine (6 x 15 mL), and the organic phase dried over anhydrous sodium

sulfate. The solvent was then evaporated under vacuum to obtain glycidyl ethers **4** (95%)**, 5** (98%), and **6** (92%).

2.3 Synthesis of hydroxyl esters 7, 9 e 10

2.3.1 Method A: Using quaternary salts of onium

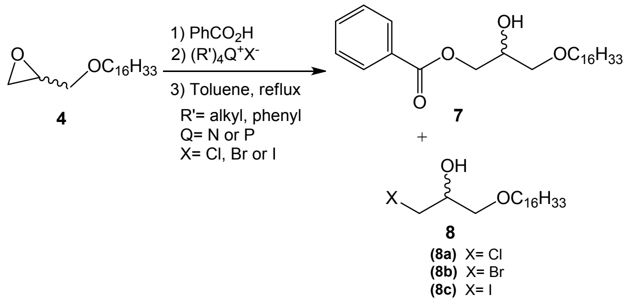

To a mixture of the epoxide (1 mmol of **4, 5, and 6**) and benzoic acid (2 mmol), methyltriphenylphosphonium iodide (1 mmol%) was added. The reaction medium was kept under stirring at 110ºC for 5 hours, and then allowed to cool to room temperature. The mixture was diluted with ethyl acetate (50 mL), washed with a satured solution of sodium bicarbonate (3 x 10 mL), and the organic phase dried over anhydrous sodium sulfate. The solvent was then evaporated under vacuum to yield **7, 9, and 10.** In the cases where previous purification through column chromatography was required, a solution of ethyl acetate/hexane 3% was used as mobile phase.

2.3.2 Method B: Using ionic liquids

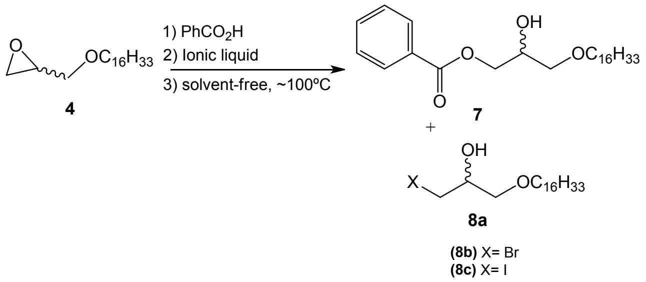

To a mixture of the epoxide (1 mmol of **4, 5, and 6**) and benzoic acid, the ionic liquid (1 mmol%) was added. The reaction mixture was kept under stirring at 110ºC and monitored by TLC until full consumption of the starting material. The mixture was allowed to cool to room temperature and diluted with distilled water (10 mL). The product was extracted with ethyl acetate (5 x 30 mL) and the organic phase dried over anhydrous sodium sulfate. The solvent was evaporated under vacuum to yield **7, 9, 10**. After isolation of the target product, the aqueous layer containing the ionic liquid was washed with ether (3x10 mL) to remove any organic impurity and dried under vacuum to recover the ionic liquid. In the cases where previous purification through column chromatography was required, a solution of ethyl acetate/hexane 3% was used as mobile phase.

2.4 Synthesis of 1-O-alkylglycerols 1-3 through "One-pot method"

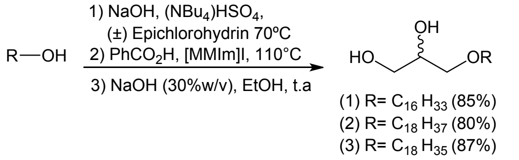

The alcohols of long aliphatic chain (1.0 mmol) were melted in a round bottom flask in the presence of tetrabutylammonium bromide (TBAB) or tetrabutylammonium hydrogen sulfate (TBAHS) (6.25 x 10^{-3} mmol) at 55ºC. The temperature was then elevated to 70°C, sodium hydroxide (1.5 mmol) was added, and the mixture was stirred for 20 minutes. Epichlorohydrin (2.0 mmol) was added to the medium under vigorous stirring. The reaction was monitored by thin layer cromatography (TLC). The reaction was quenched when the amount of product formed was kept constant. Then, benzoic acid (2 mmol) and the ionic liquid [MMIm]I (1 mmol%) were added. The reaction mixture was kept under stirring at 110 ºC and monitored by TLC until full consumption of the starting material. The mixture was allowed to cool to room temperature, diluted with a solution of sodium hydroxide (2.5 mmol: 30% NaOH) and ethanol (50 mL), and kept under stirring at room temperature for 10 h. The product was extracted with ethyl acetate (50 mL) and washed with distilled water (6 x 10 mL). The solvent was evaporated under vacuum and the residue was purified through column chromatography eluting with ethyl acetate/hexane (20%) to give the 1-O-alkylglycerols **1** (85%), **2** (80%), and **3** (87%).

3. The 1D NMR spectra of 1-10

Chimyl alcohol (1): 100% yield (1.17g), white solid, (mp. 62-64°C). **IR** (KBr, ν_{max}/cm^{-1}): 3368 (OH), 2954 – 2850 (CH), 1471 (CH), 1.124 (C-O). **^{1}H NMR** (300 MHz, $CDCl_3$) δ 3.87 (m, 2H), 3.73 (m, 3H), 3.50 (m, 4H), 1.58 (m, 4H), 1.26 (s, 24H), 0.89 (t, J= 6.0, 3H). **^{13}C NMR** (100 MHz, $CDCl_3$) δ 72.02, 71.41, 70.02, 63.82, 31.46, 25.62, 13.65.

Batyl alcohol (2): 100% yield (1.15g); white solid (mp. 70-72°C). **IR** (KBr, ν_{max}/cm^{-1}) : 3363 (OH), 2954 – 2850 (CH), 1471 (CH), 1.123 (C-O). **^{1}H NMR** (300 MHz, $CDCl_3$) δ 3.87 (m, 2H), 3.73 (m, 3H), 3.65 (m, 4H), 1.58 (d, J= 6Hz, 4H), 1.26 (s, 26H); 0.89 (t, J= 6.6 Hz, 3H). **^{13}C NMR** (100 MHz, $CDCl_3$) δ 72.04, 71.41, 69.99, 63.83, 31.47, 25.62, 13.65.

Selachyl alcohol (3): 100% yield (1.10g); colorless oil. **IR** (KBr, ν_{max}/cm^{-1}) : 3383 (OH), 3004 – 2854 (CH), 1732 – 1654 (C=C), 1464 (CH), 1120 (C-O). **^{1}H NMR** (300 MHz, $CDCl_3$) δ 5.36 (m, 2H), 3.86 (m, 2H), 3.67 (m, 3H), 3.48 (m, 4H), 2.03(d, J= 6Hz, 4H), 1.59 (m, 6H), 1.28 (d, J= 6Hz, 18H), 0.89 (t= 6.0, J= 6Hz, 3H). **^{13}C NMR** (100 MHz, $CDCl_3$) δ 129.52, 72.06, 71.40, 69.97, 63.84, 31.45, 28.86, 26.76, 25.62, 22.23, 13.66.

1-*O*-Hexadecyl-2,3-epoxypropane (4): 95% yield (1.17 g), white solid, (mp. 24-26ºC). **IR** (KBr, ν_{max}/cm^{-1}): 3048- 2851 (CH), 1467 (CH) , 1253 (C-O), 1114 (C-O), 906 (C-C), 852 (C-C). **^{1}H NMR** (300 MHz, $CDCl_3$) δ 3.72 (dd, J = 11.6 e 3.1 Hz, 2H), 3.51 – 3.35 (m, 2H), 3.18 – 3.13 (m, 1H), 2.79 (t, J = 9

Hz, 1H), 2.61 (dd, J = 4.9 e 2.9 Hz, 1H), 1.60 – 1.56 (m, 2H), 1.25 (s, 26H); 0.88 (t, J = 6.6 Hz, 3H). **^{13}C NMR** (100 MHz, $CDCl_3$) δ 72.1, 71.8, 51.3, 44.7, 32.3, 30.8, 29.9, 29.7, 29.5, 23.1, 14.5.

1-*O*-Octadecyl-2,3-epoxypropane (5): 98% yield (1.18g); white solid, (mp. 42-45°C). **IR** (KBr, ν_{max}/cm^{-1}): 3052 (CH), 3000- 2850 (CH), 1473-1378 (CH), 1251 (C-O), 1125 (C-O), 906 (CH), 852 (CH), 729 (CH). **^{1}H NMR** (500 MHz, $CDCl_3$) δ 3.72 (dd, J = 11.6 e 3.1 Hz, 2H), 3.53 – 3.37 (m, 2H), 3.18 – 3.15 (m, 1H), 2.80 (t, J = 5 Hz, 1H), 2.61 (dd, J = 5 and 5 Hz, 1H), 1.64 – 1.57 (m, 2H), 1.26 (s, 30H); 0.88 (t, J = 6.6 Hz, 3H). **^{13}C NMR** (100 MHz, $CDCl_3$) δ 71.5, 71.2, 50.6, 44.1, 31.6, 30.8, 29.4, 29.1, 25.8, 22.4, 13.8.

1-*O*-Oleyl-2,3- epoxy propane (6): 92% yield (1.11 g); colorless oil. **IR** (KBr, ν_{max}/cm^{-1}): 3052 (CH), 3002-2854 (CH), 1732 – 1655 (C=C), 1465 (CH), 1253 (C-O), 1125 (C-O). **^{1}H NMR** (300 MHz, $CDCl_3$) δ 5,36 (m, 2H); 3,72 (dd, J = 11.5 and 3.2 Hz, 2H); 3.52 – 3,36 (m, 2H), 3.19 – 3.14 (m, 1H), 2.80 (t, J = 9 Hz, 1H), 2.62 (dd, J = 4.9 and 2.9 Hz, 1H), 2.02 – 1.98 (m, 4H), 1.63 – 1.54 (m, 2H), 1.30 (d, J= 6, 22H), 0.88 (t, J = 6.6 Hz, 3H). **^{13}C NMR** (100 MHz, $CDCl_3$) δ 129.5, 129.5, 72.1, 71.9, 51.3, 44.7, 32.3, 30.1, 29.7, 29.6, 27.6, 26.5, 23.1, 14.5.

2-Hydroxy-3-(hexadecyloxy)propyl benzoate (7): 95% yield (1.57g), yellowish oil. **IR** (KBr, ν_{max}/cm^{-1}): 3519 (OH), 2923 – 2853 (CH), 1721 (C=O), 1.120 (C-O).**^{1}H NMR** (500 MHz, $CDCl_3$): δ 8.05 (d, J=8.5, 2H), δ 7.56 (t, J= 15, 1H), 7.44 (t, J= 15,5, 2H), δ 4.40 (m, 1H), δ 4.14 (m, 1H), δ 3.72 (m, 1H), δ 3.58 – 3.45 (m, 4H), δ 1.60 – 1.56 (m, 4H), δ 1.29 (s, 24H), 0.88 (t, J = 14 Hz, 3H). **^{13}C NMR** (100 MHz, $CDCl_3$): 166.67, 133.15, 129.89, 129.79, 129.71, 128.41, 73.74, 71.82, 68.96, 66.07, 31.94, 29.71, 26.10, 22.71, 14.14.

1-Chloro-3-(hexadecyloxy)propan-2-ol (8a): yellowish oil. **IR** (KBr, ν_{max}/cm^{-1}): 3406 (OH), 2922 – 2852 (CH), 1.120 (C-O). **^{1}H NMR** (400 MHz, $CDCl_3$): δ 3.94 (m, 1H), 3.56 (m, 4H), 3.41 (m, 2H), 1.57 (m, 4H), 1.30 (s, 24H), 0.88 (t, J= 8.0, 3H);
^{13}C NMR (100 MHz, $CDCl_3$): δ 72.50, 71.76, 70.25, 64.28, 62.07, 46.00, 34.21, 31.91, 30.30, 29.67, 29.58, 29.34, 26.03, 22.67, 14.11.

1-Bromo-3-(hexadecyloxy)propan-2-ol (8b): brownish oil. **IR** (KBr, ν_{max}/cm^{-1}): 3417 (OH), 2924 – 2853 (CH), 1.120 (C-O). **^{1}H NMR** (400 MHz, $CDCl_3$): δ 3.94 (m, 1H), 3.56 (m, 4H), 3.41 (m, 2H), 1.57 (m, 4H), 1.30 (s, 24H), 0.88 (t, J= 8.0, 3H).**^{13}C NMR** (100 MHz, $CDCl_3$): δ 72.01, 71.96, 70.13, 66.90, 66.61, 50.16, 33.71.46, 29.88, 22.92, 14.37.

1-Iodo-3-(hexadecyloxy)propan-2-ol (8c): brownish oil. **IR** (KBr, ν_{max}/cm^{-1}): 3441 (OH), 2920 – 2851 (CH), 1.110 (C-O). **^{1}H NMR** (400 MHz, $CDCl_3$): δ 4.00 (m, 1H), 3.65 (m, 4H), 3.53 (m, 2H), 1.58 (m, 4H), 1.30 (s, 24H), 0.89 (t, J= 12.0, 3H). **^{13}C NMR** (100 MHz, $CDCl_3$): δ 73.18, 72.35, 70.36, 69.44, 63.92, 33.71.46, 29.88, 22.92, 14.37, 9.82, 7.00.

2-Hydroxy-3-(octadecyloxy)propyl benzoate (9): 95% yield (1.54g), yellowish oil. **IR** (KBr, ν_{max}/cm^{-1}): 3426 (OH), 2920 – 2851 (CH), 1721 (C=O), 1.117 (C-O). **^{1}H NMR** (400 MHz, $CDCl_3$): δ 8.08 (d, J= 12.8, 2H), δ 7.60 (t, J= 14.4, 1H), 7.46 (t, J= 15.2, 2H), δ 4.42 (m, 1H), δ 4.15 (m, 1H), δ 3.60 (m, 1H), δ 3.59 – 3.48 (m, 4H), δ 1.60 – 1.57 (m, 4H), δ 1.26 (s, 28H); 0.88 (t, J = 13.2, 3H). **^{13}C NMR** (100 MHz, $CDCl_3$) 166.64, 133.51, 133.12, 130.14, 130.02, 129.93, 129.79, 129.70, 128.39, 73.77, 71.51, 68.98, 66.07, 63.04, 31.93, 29.70, 26.09, 22.69, 14.10.

(Z)-2-Hydroxy-3-(octadec-9-enyloxy)propyl benzoate (10): 95% yield (1.43g), yellowish oil. **IR** (KBr, ν_{max}/cm^{-1}): 3434 (OH), 2925 – 2854 (CH), 1722 (C=O), 1619 (C=C), 1.117 (C-O). **^{1}H NMR** (500 MHz, $CDCl_3$): δ 8.07 (d, J= 9.5, 2H), δ 7.58 (t, J= 7, 1H), 7.47 (t, J= 15.5, 2H), δ 5,36 (m, 2H); δ 4.42 (m, 1H), δ 4.15 (m, 1H), δ 3.59 (m, 1H), δ 3.58 – 3.48 (m, 4H), δ 2.02 – 1.99 (m, 4H), 1.60 – 1.53 (m, 4H), 1.33 (s, 20H), 0.88 (t, J =13.5, 3H). **^{13}C NMR** (100 MHz, $CDCl_3$): 166.64, 133.15, 133.11, 129.95, 129.83, 129.78, 129.70, 128.39, 73.77, 71.50, 68.99, 66.07, 63.12, 32.59, 31.90, 29.69, 26.08, 22.67, 14.09.

4. ESI-HRMS analysis for the 7-10

ESI- HRMS m/z: $[M]^+$ Calcd for $C_{26}H_{44}O_4$ 420.3240; Found 420.3312.

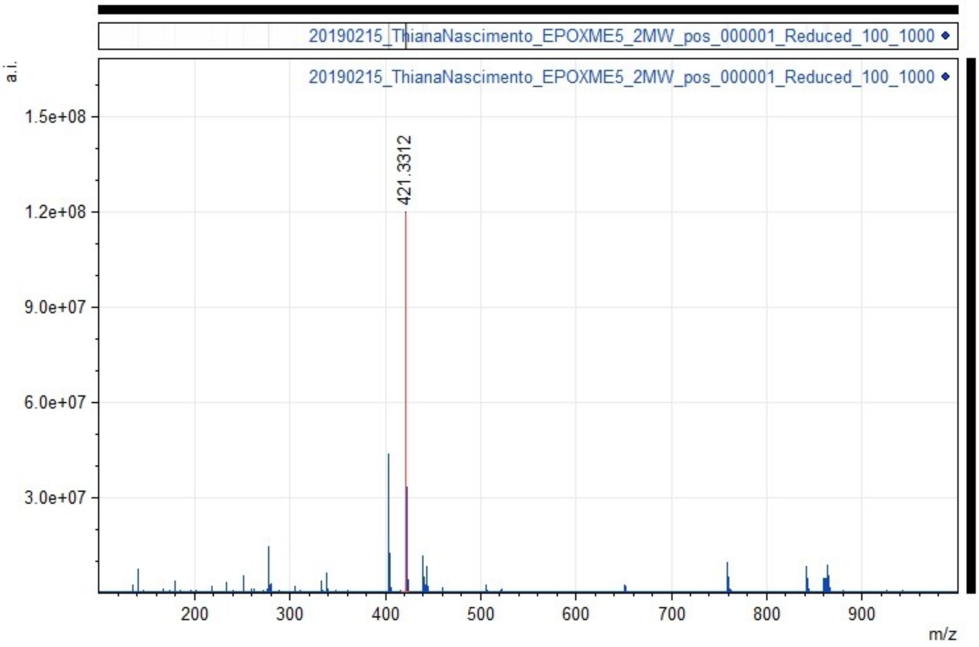

Figure S1. Mass spectrum for **7**

ESI-HRMS m/z: $[M + Na]^+$ Calcd for $C_{19}H_{39}ClO_2$ 334.2639 ; Found 334.2531

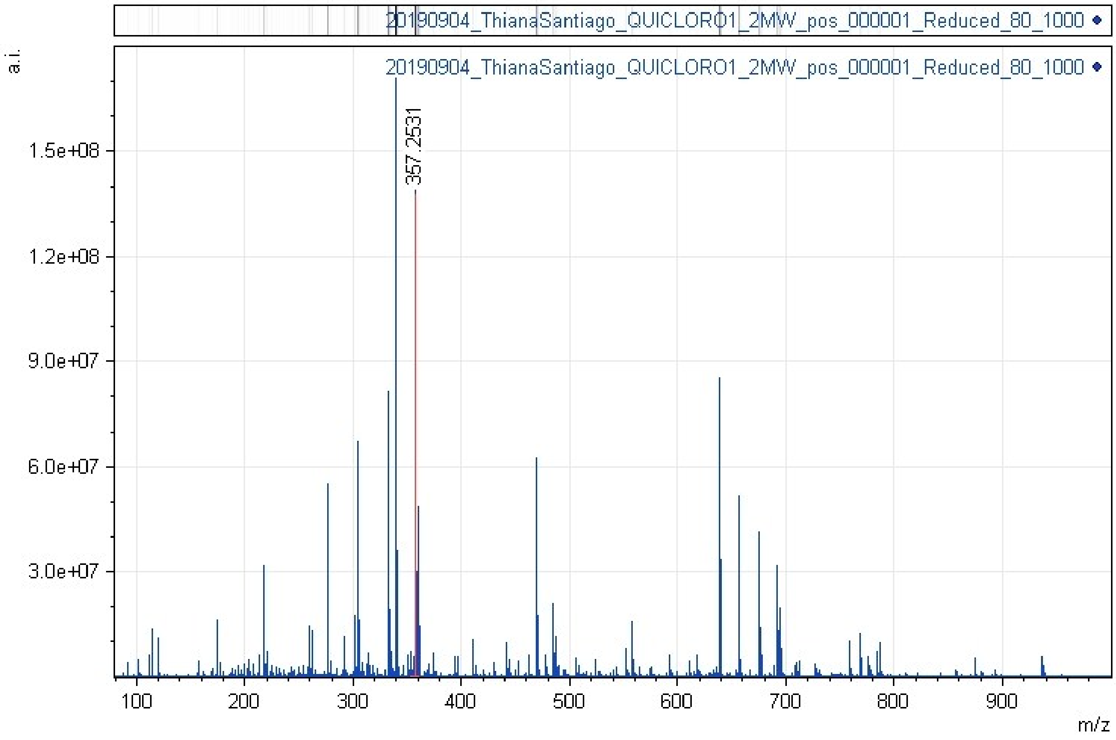

Figure S2. Mass spectrum for **8a**

ESI-HRMS m/z: [M + Na]$^+$ Calcd for $C_{19}H_{39}BrO_2$ 378.2133; Found 378.2026.

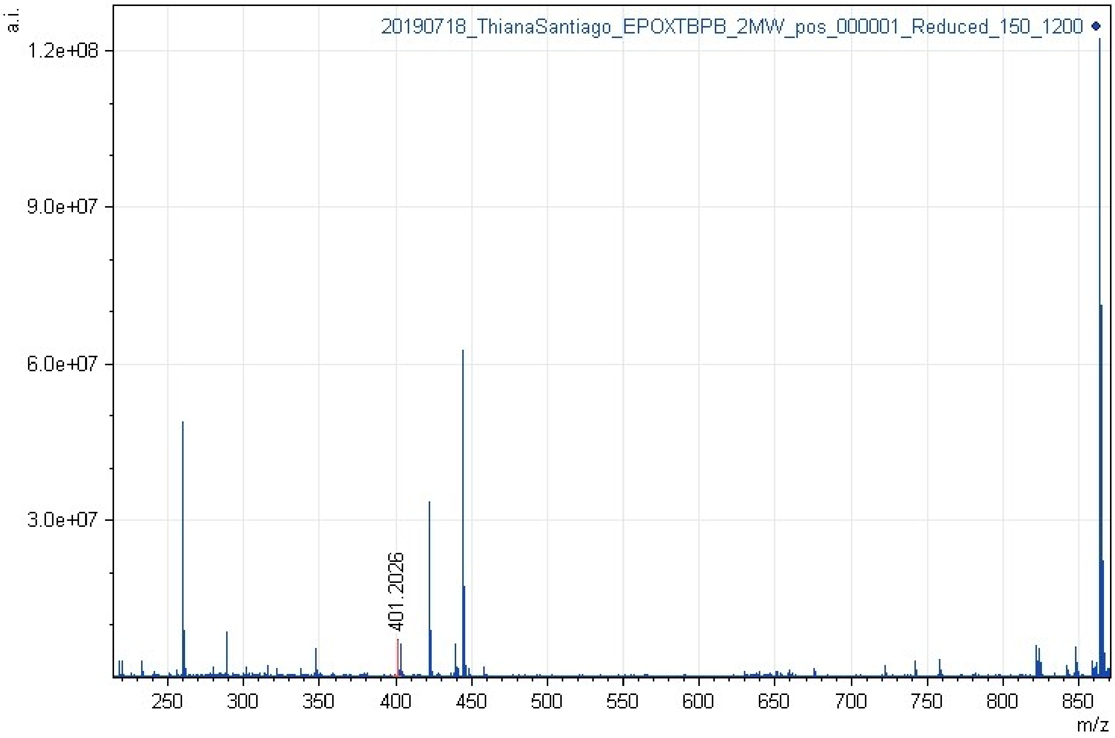

Figure S3. Mass spectrum for **8b**

ESI-HRMS m/z: [M + Na]$^+$ Calcd for $C_{19}H_{39}IO_2$ 426.1995; Found 426.1884.

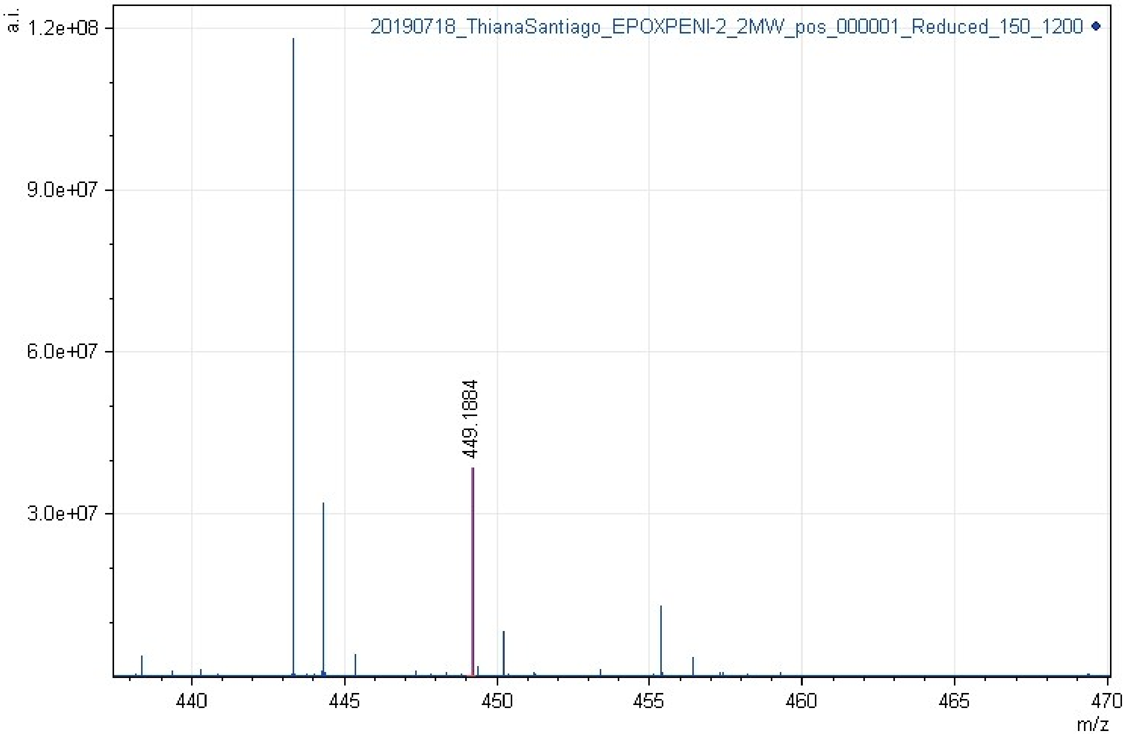

Figure S4. Mass spectrum for **8c**

ESI-HRMS m/z: $[M]^+$ Calcd for $C_{28}H_{48}O_4$ 448.3553; Found 448.3625

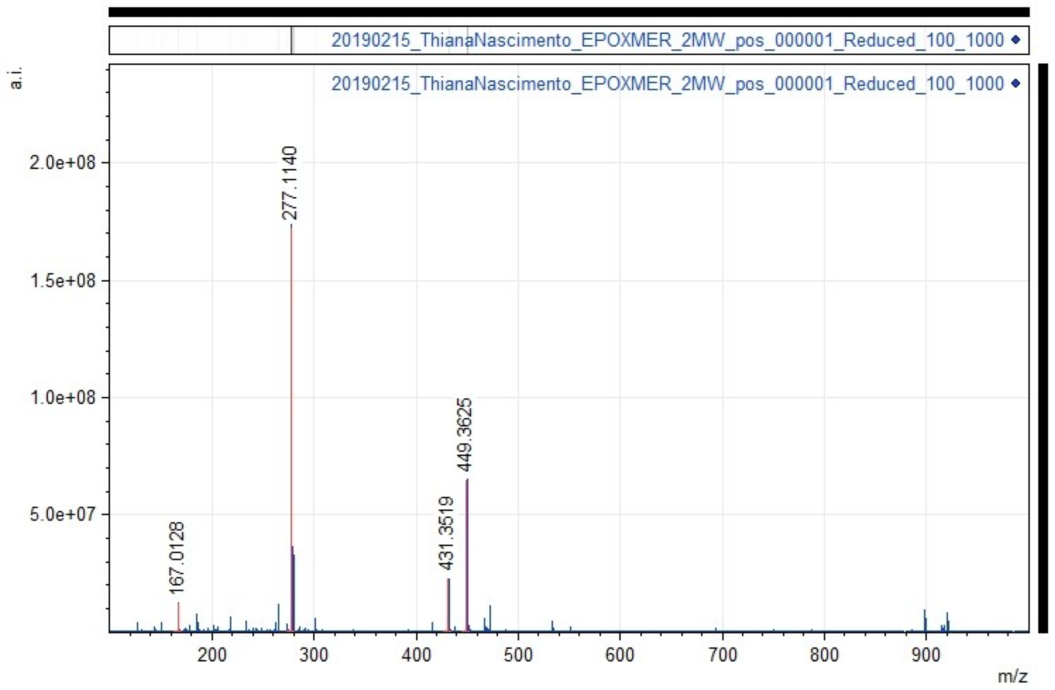

Figure S5. Mass spectrum for **9**

ESI-HRMS m/z: $[M + Na]^+$ Calcd for $C_{28}H_{46}O_4$ 446.3396; Found 446.3286

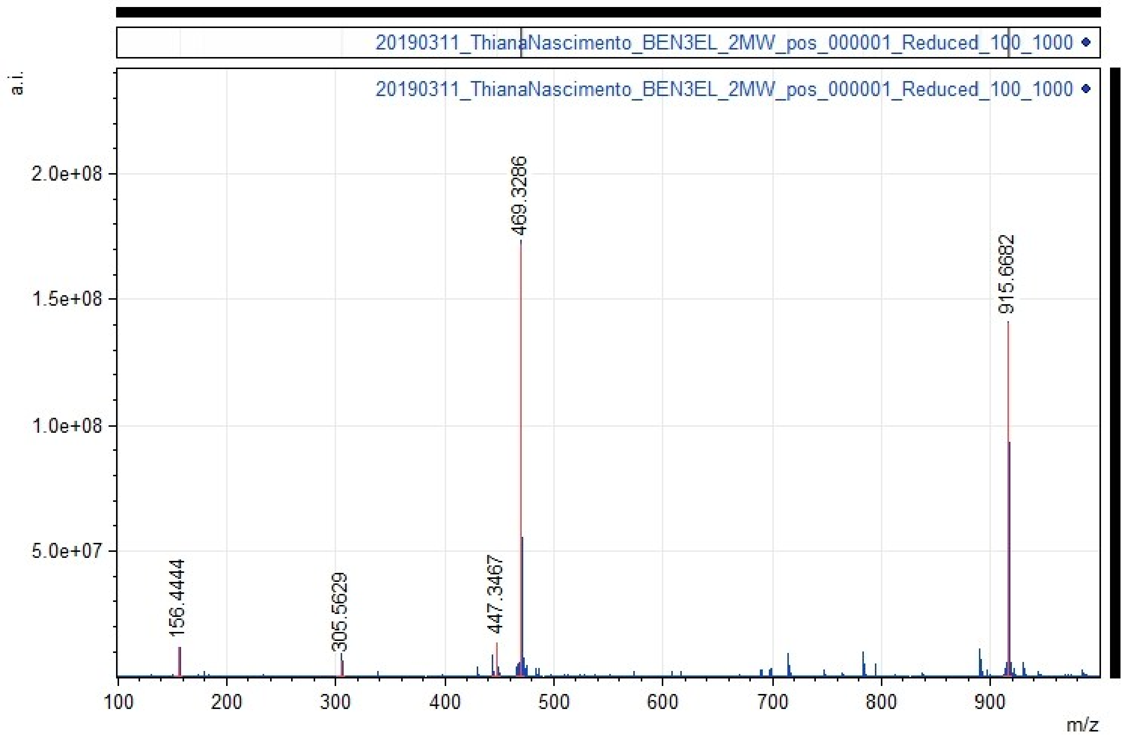

Figure S6. Mass spectrum for **10**

5. Copies of 1D NMR spectra of 1-10

^{1}H (300 MHz, 400 MHz or 500) and ^{13}C (100 MHz) NMR spectra were recorded on an NMR spectrometer in $CDCl_3$. Chemical shifts (δ) are reported in parts per million downfield from tetramethylsilane (TMS δ = 0.00).

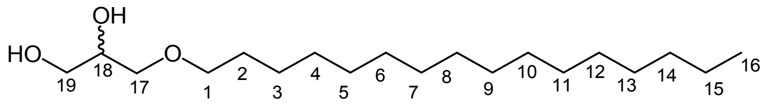

1

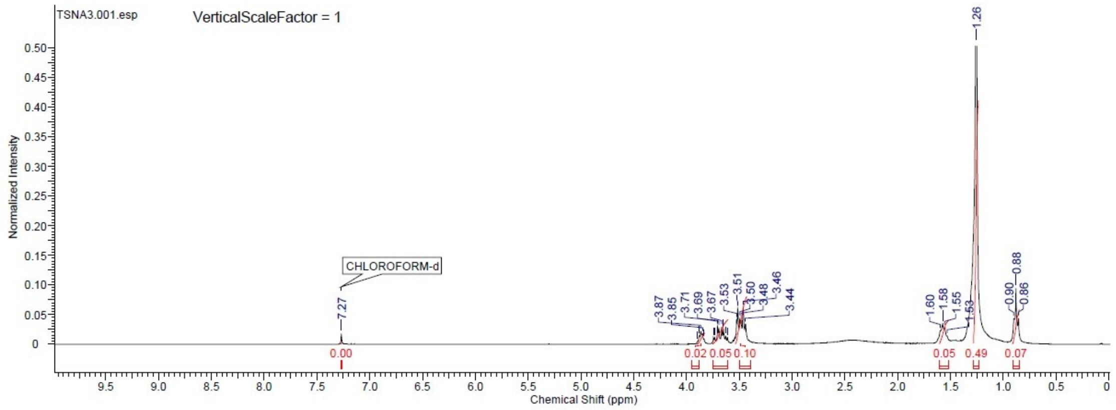

Figure S7. ^{1}H NMR (300 MHz, $CDCl_3$) spectrum for **1**

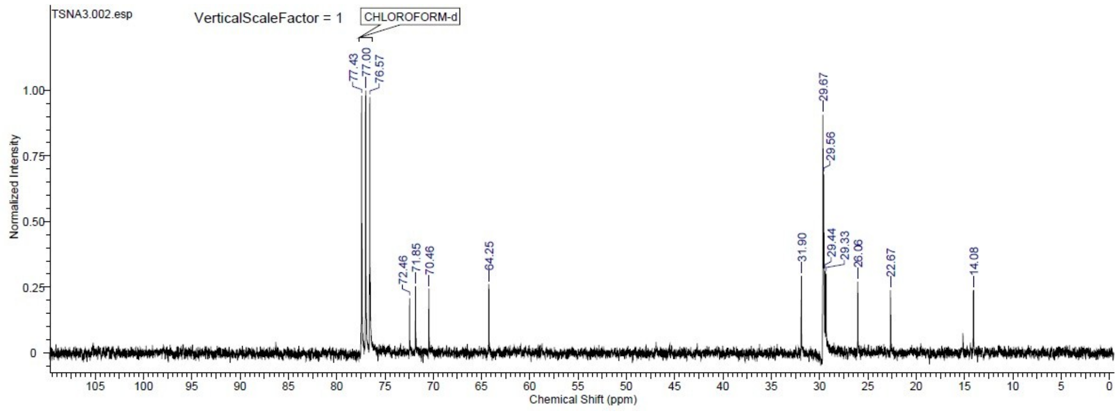

Figure S8. ^{13}C NMR (100 MHz, $CDCl_3$) spectrum for **1**

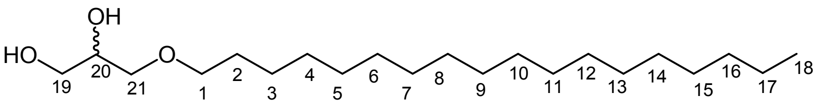

2

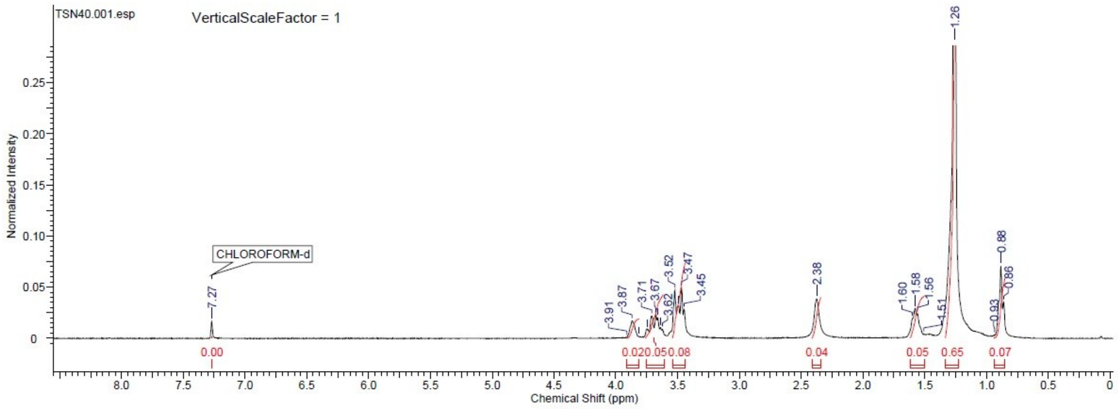

Figure S9. ${}^{1}H$ NMR (300 MHz, $CDCl_3$) spectrum for **2**

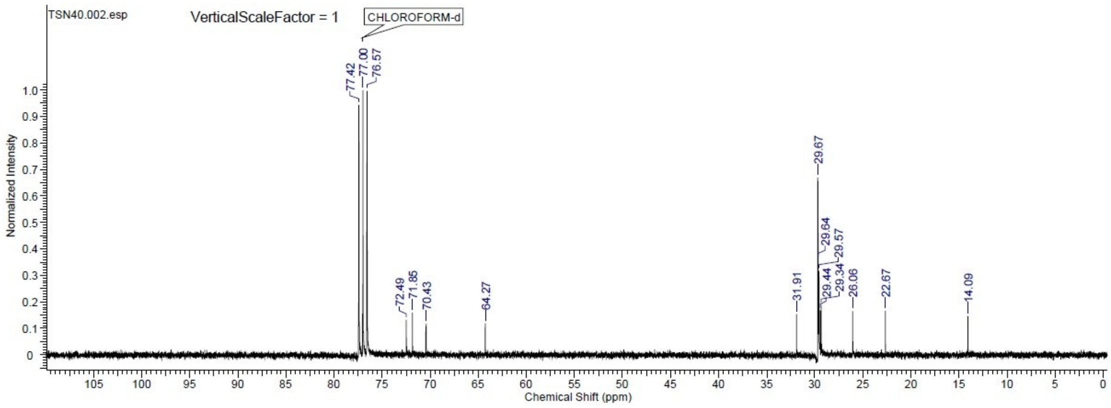

Figure S10. ${}^{13}C$ NMR (100 MHz, $CDCl_3$) spectrum for **2**

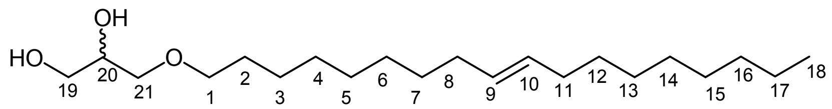

3

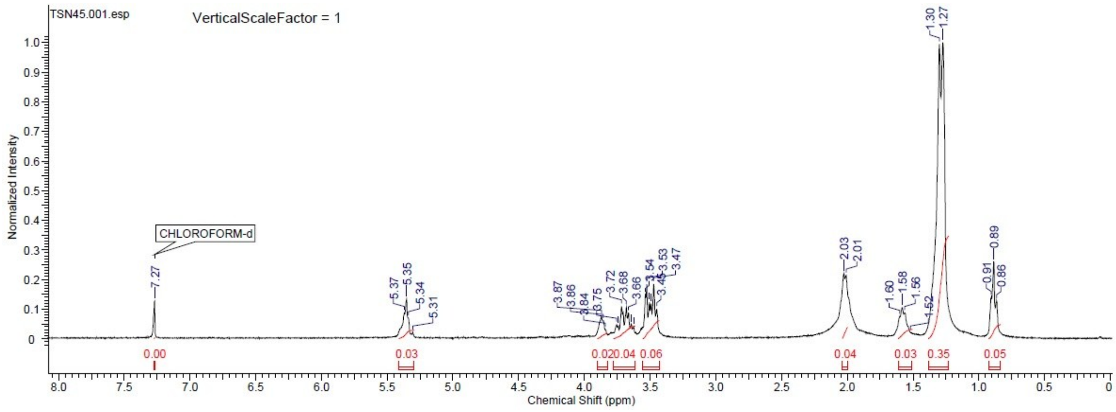

Figure S11. ^{1}H NMR (300 MHz, $CDCl_3$) spectrum for **3**

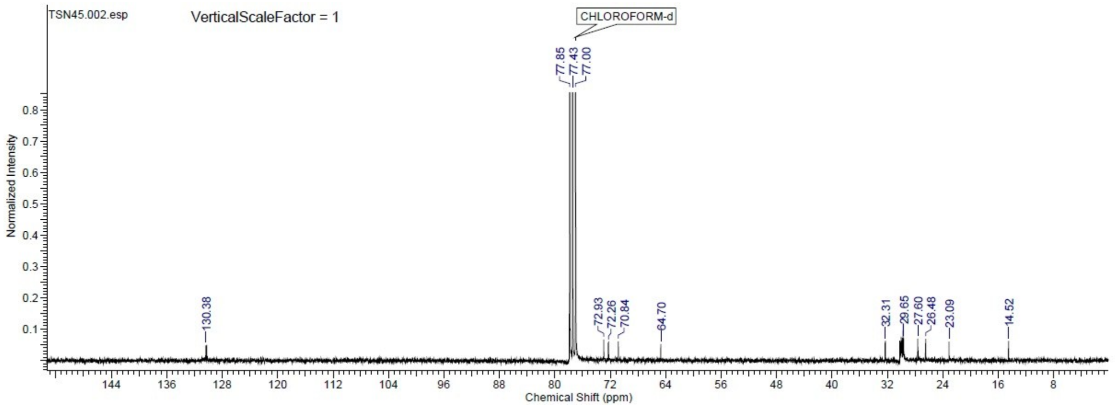

Figure S12. ^{13}C NMR (100 MHz, $CDCl_3$) spectrum for **3**

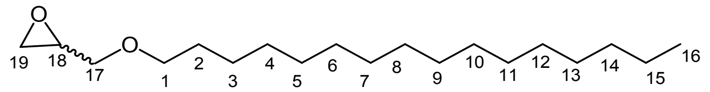

4

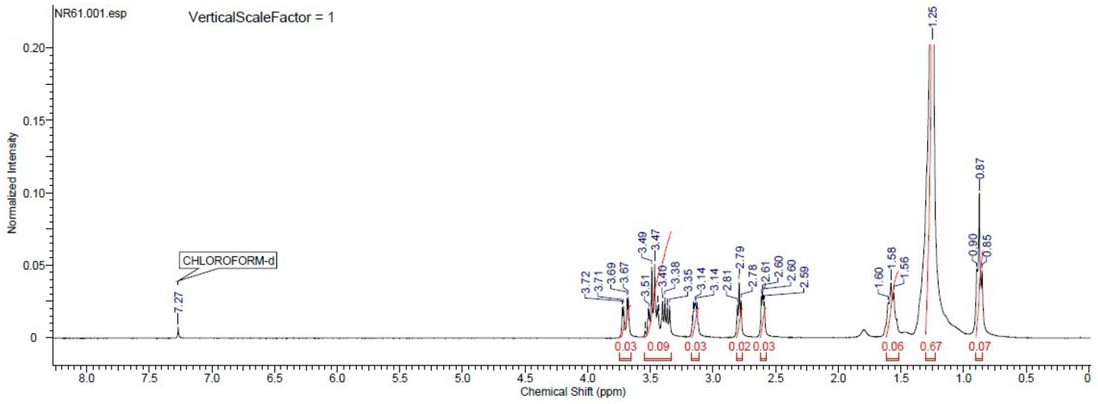

Figure S13. ^{1}H NMR (300 MHz, $CDCl_3$) spectrum for **4**

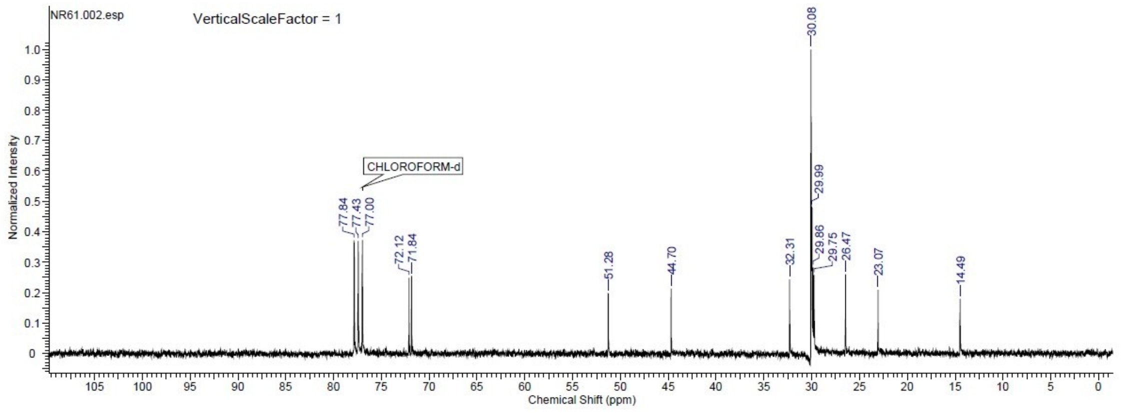

Figure S14. ^{13}C NMR (100 MHz, $CDCl_3$) spectrum for **4**

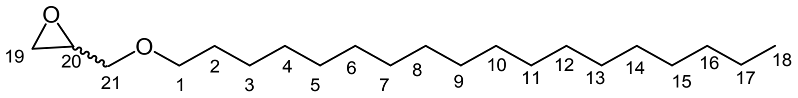

5

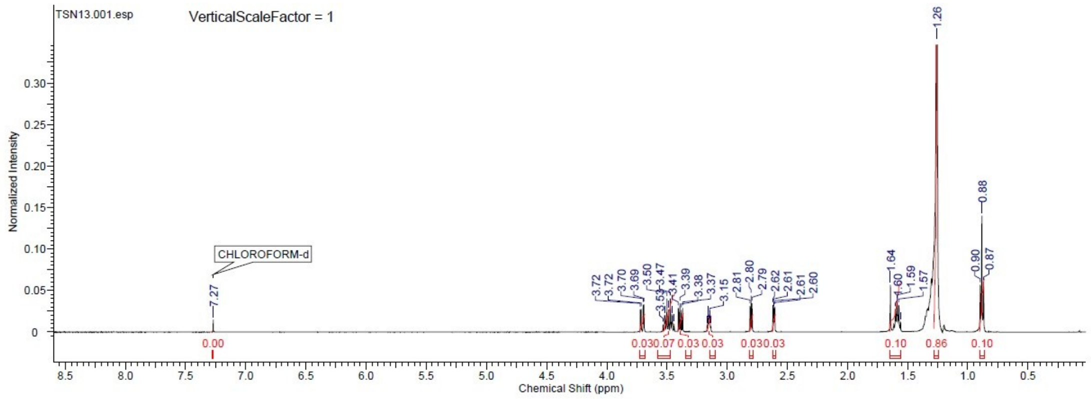

Figure S15. ^{1}H NMR (500 MHz, $CDCl_3$) spectrum for **5**

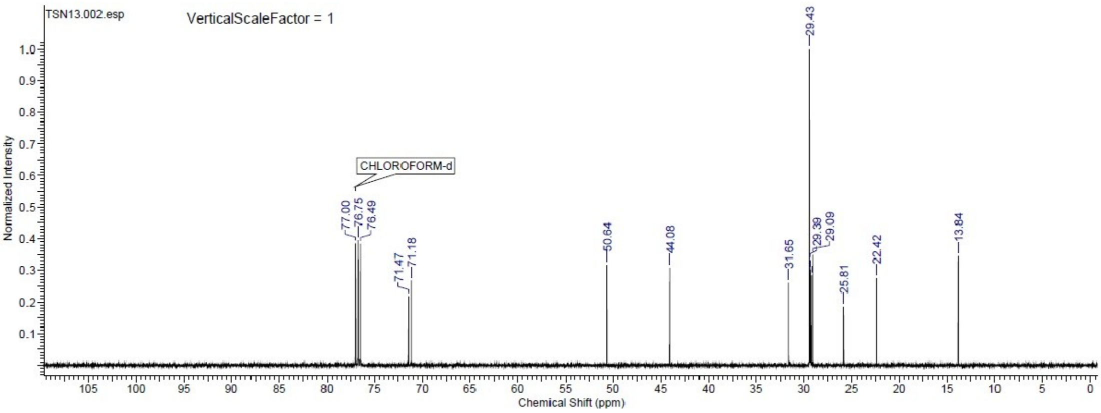

Figure S16. ^{13}C NMR (100 MHz, $CDCl_3$) spectrum for **5**

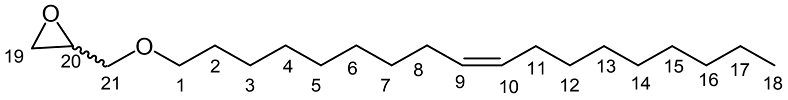

6

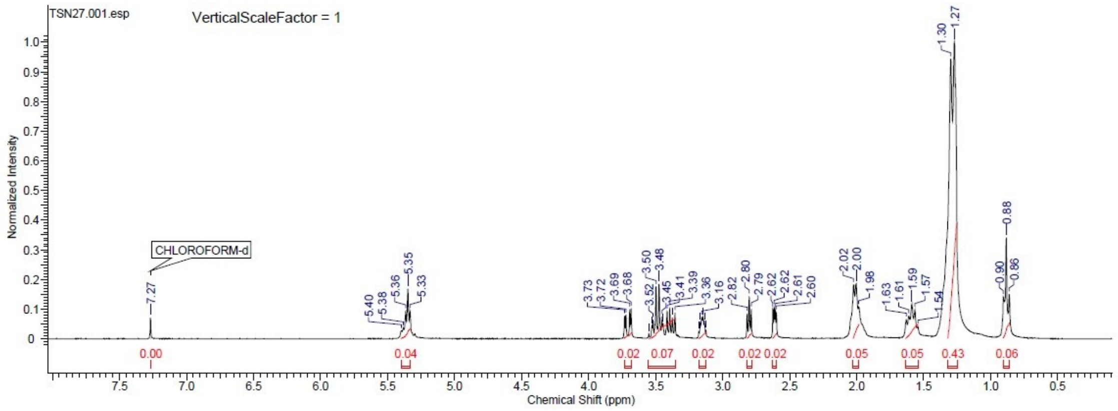

Figure S17. ^{1}H NMR (300 MHz, $CDCl_3$) spectrum for **6**

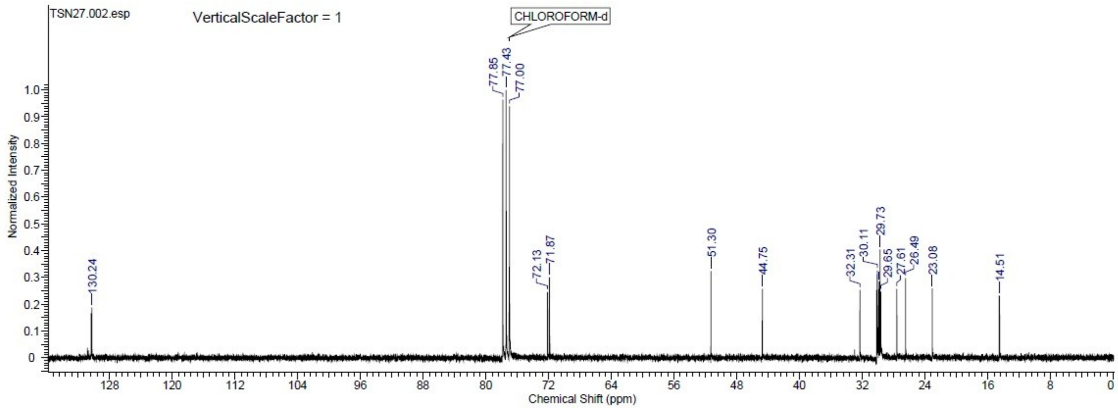

Figure S18. ^{13}C NMR (100 MHz, $CDCl_3$) spectrum for **6**

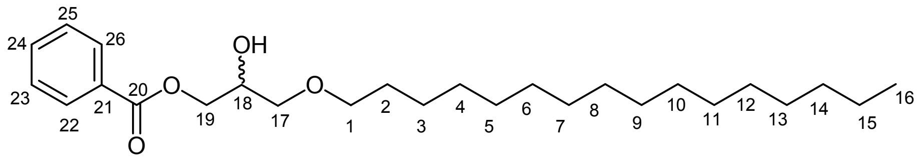

7

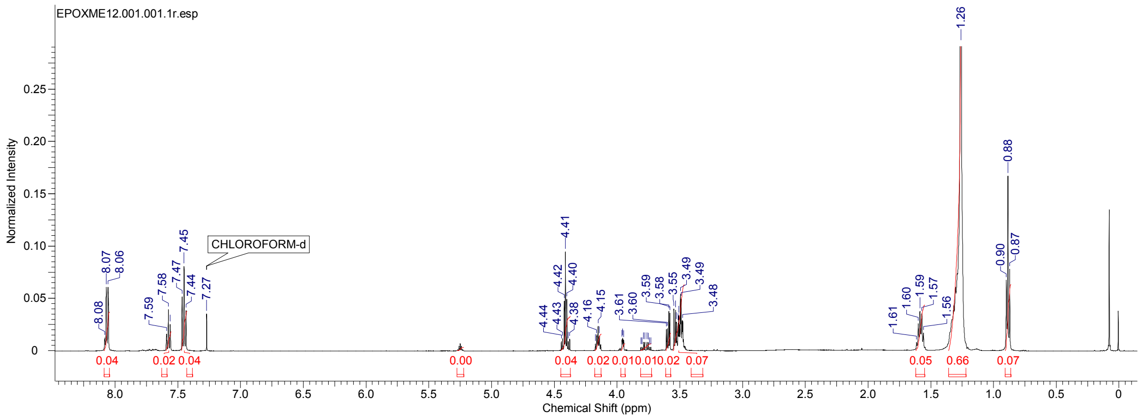

Figure S19. ^{1}H NMR (500 MHz, $CDCl_3$) spectrum for **7**

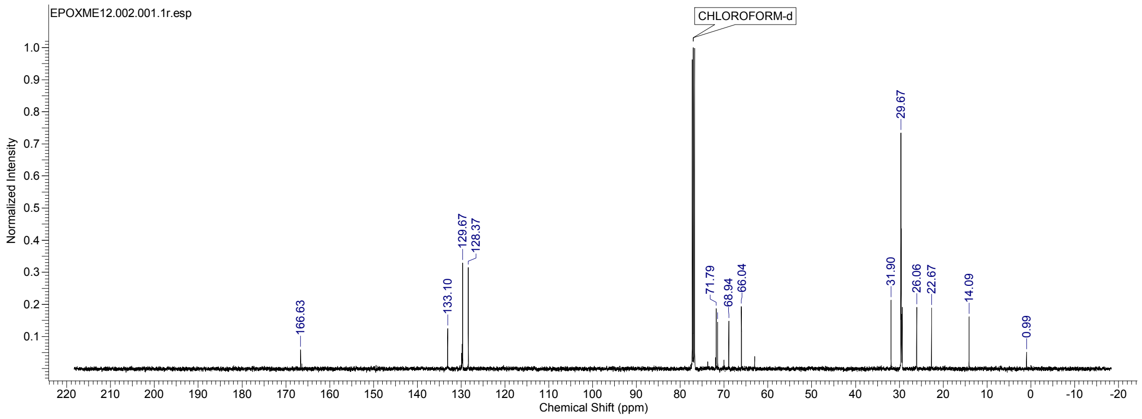

Figure S20. ^{13}C NMR (125 MHz, $CDCl_3$) spectrum for **7**

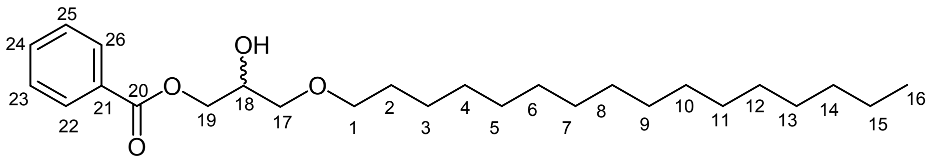

7

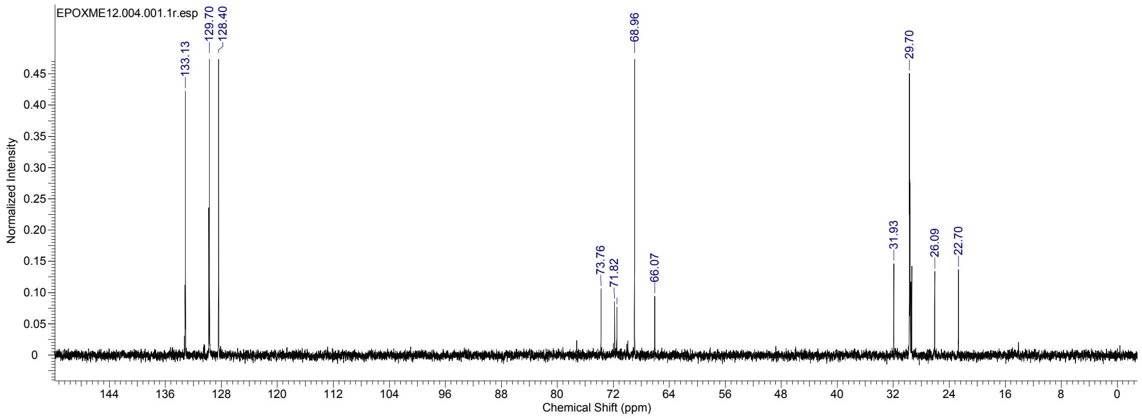

Figure S21. DEPT-90 NMR (125 MHz, $CDCl_3$) spectrum for **7**

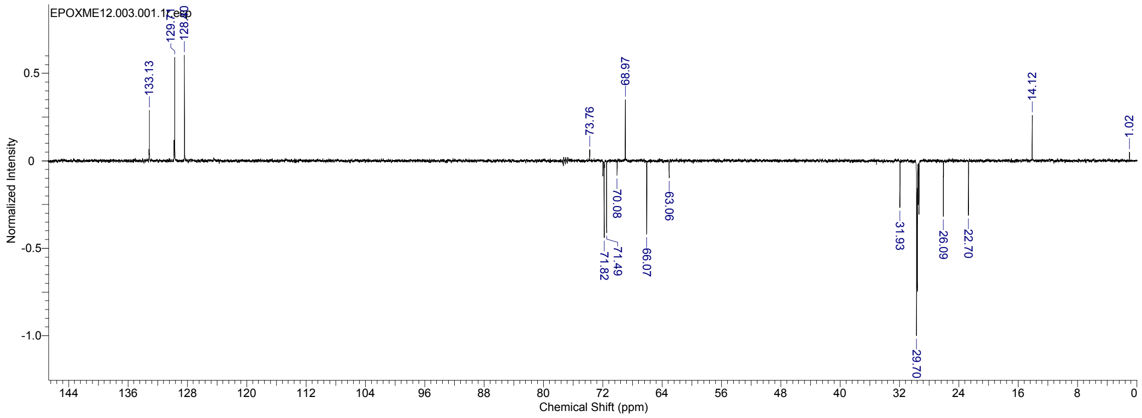

Figure S22. DEPT-135 NMR (125 MHz, $CDCl_3$) spectrum for **7**

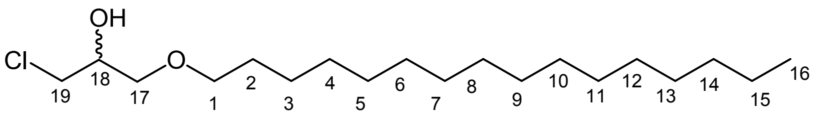

8a

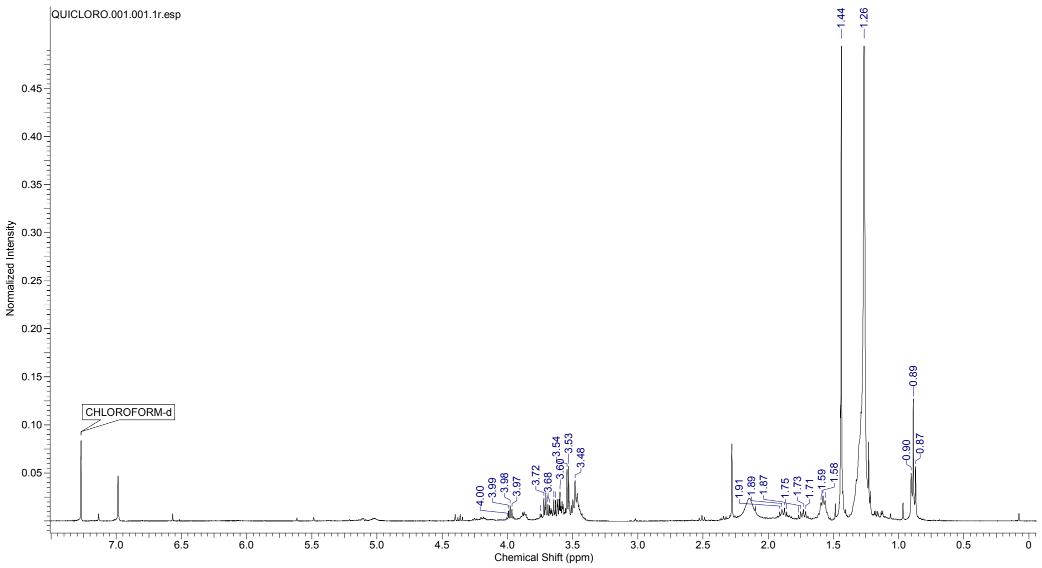

Figure S23. ^{1}H NMR (400 MHz, $CDCl_3$) spectrum for **8a**

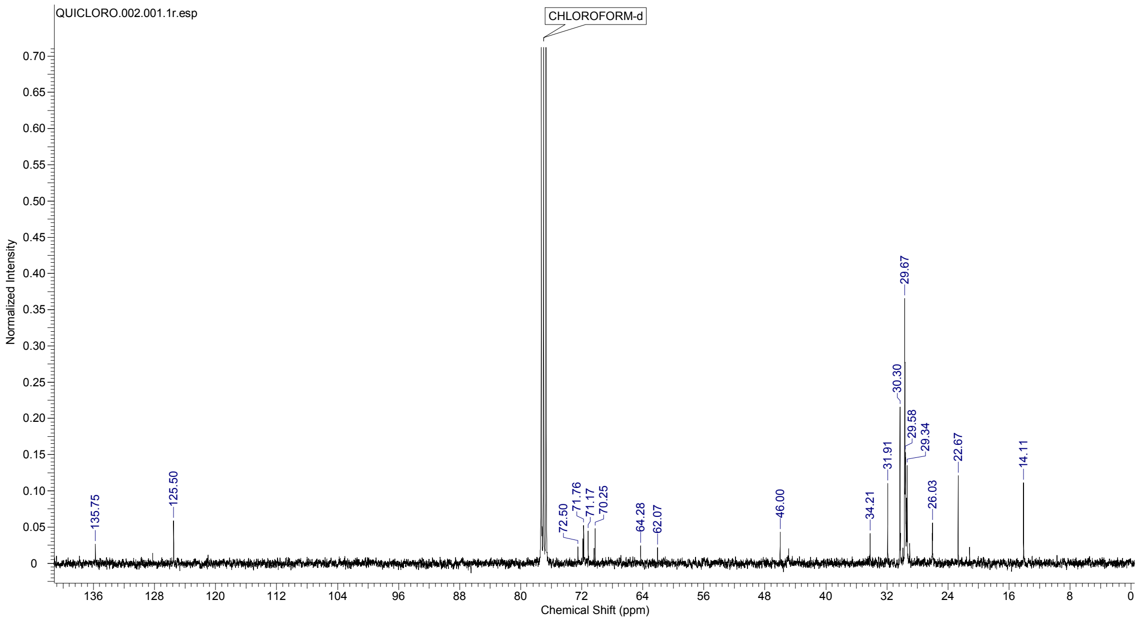

Figure S24. ^{13}C NMR (100 MHz, $CDCl_3$) spectrum for **8a**

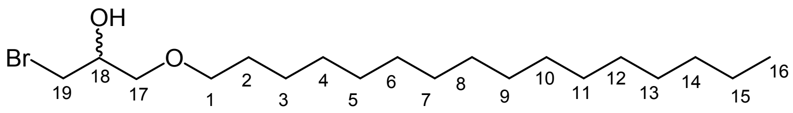

8b

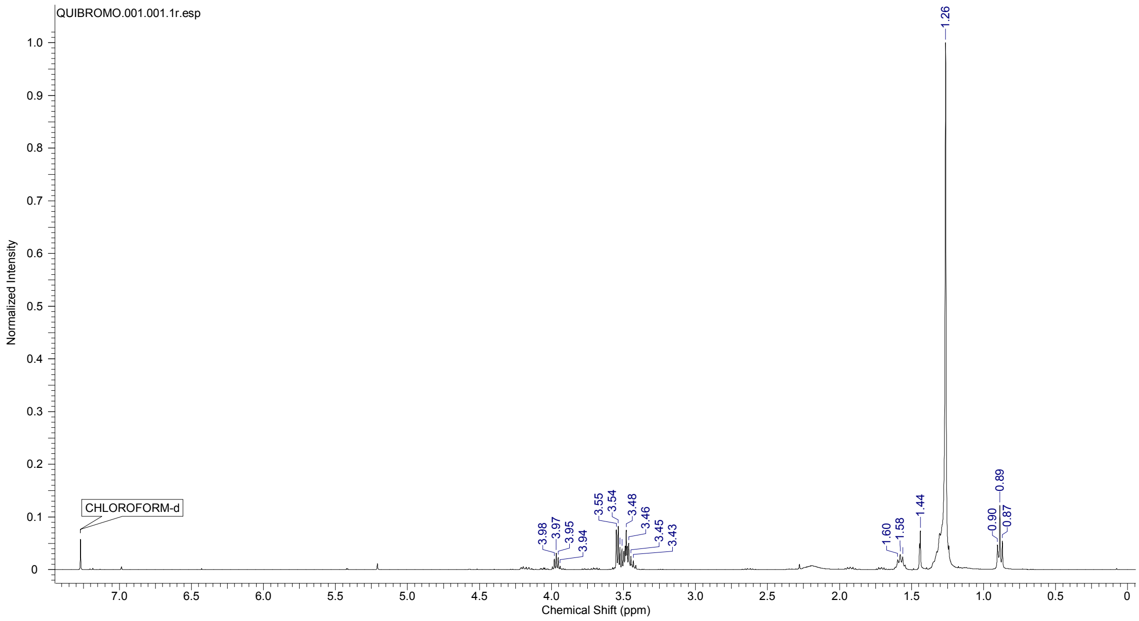

Figure S25. ^{1}H NMR (400 MHz, $CDCl_3$) spectrum for **8b**

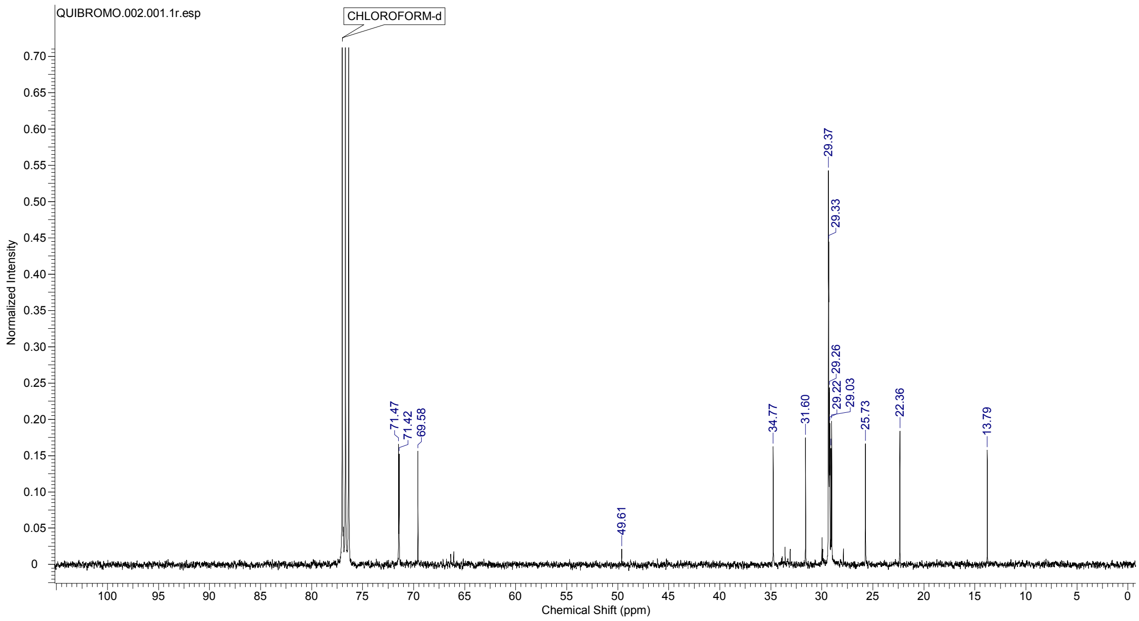

Figure S26. ^{13}C NMR (100 MHz, $CDCl_3$) spectrum for **8b**

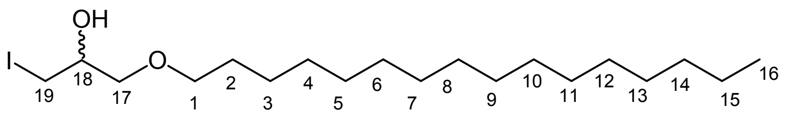

8c

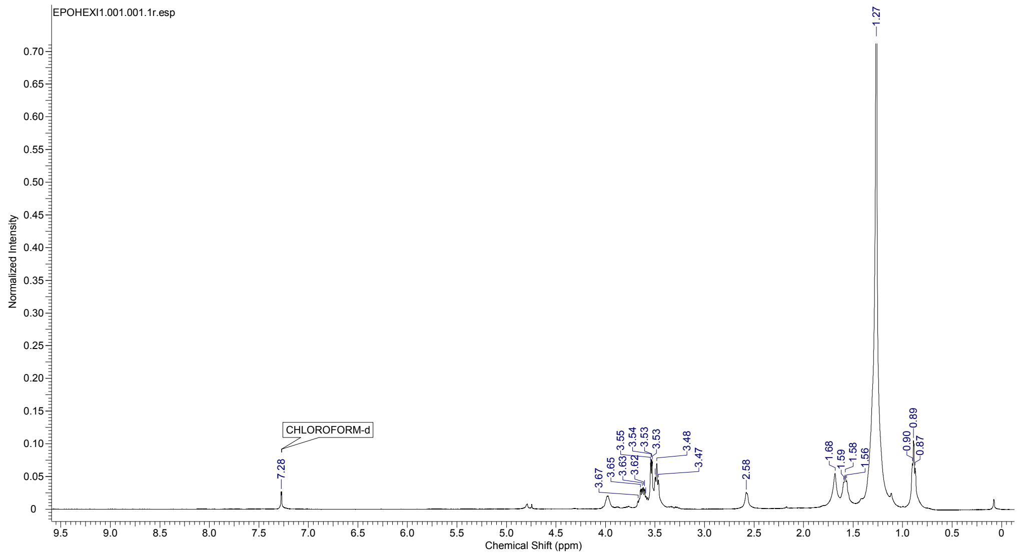

Figure S27. ^{1}H NMR (400 MHz, $CDCl_3$) spectrum for **8c**

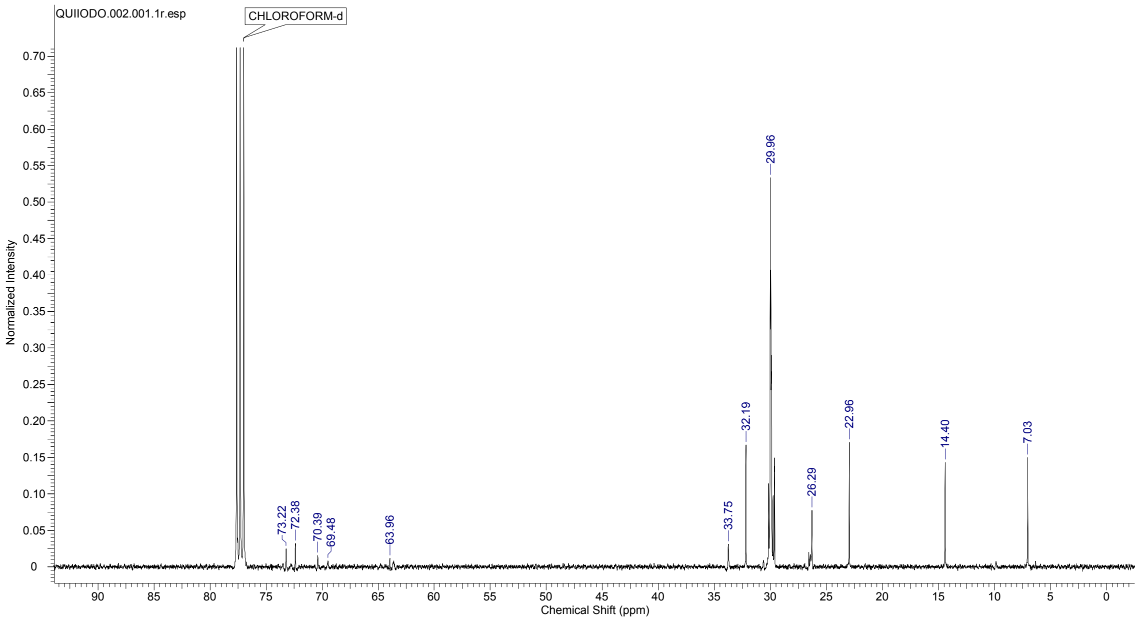

Figure S28. ^{13}C NMR (100 MHz, $CDCl_3$) spectrum for **8c**

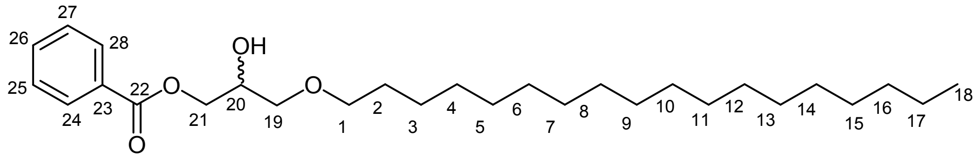

9

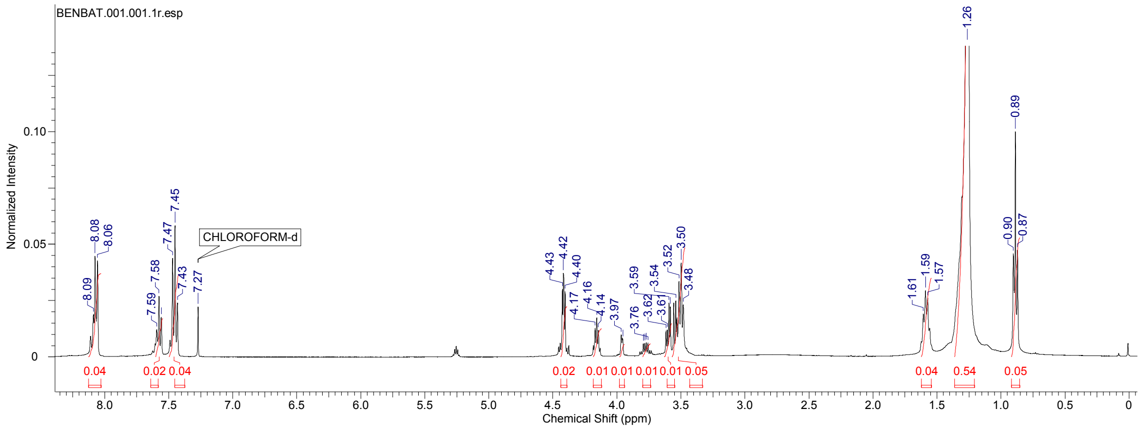

Figure S29. ^{1}H NMR (400 MHz, $CDCl_3$) spectrum for **9**

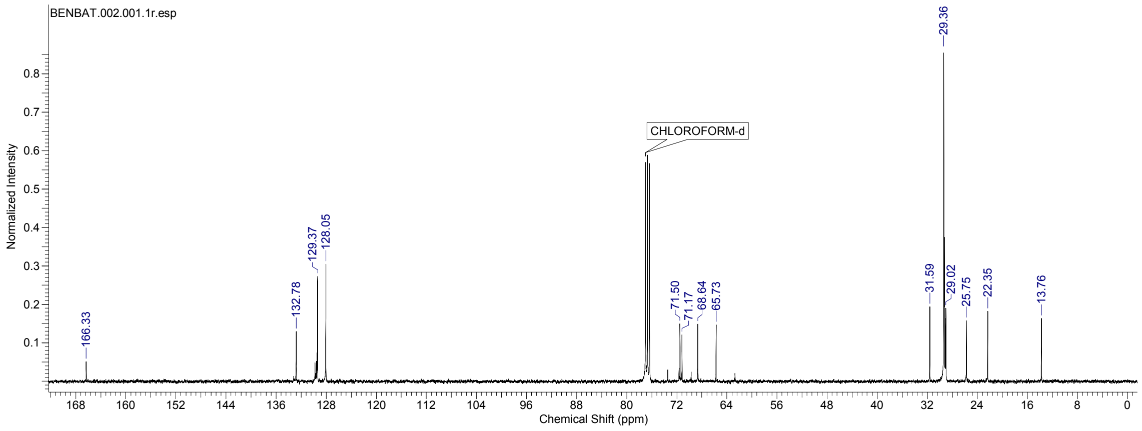

Figure S30. ^{13}C NMR (100 MHz, $CDCl_3$) spectrum for **9**

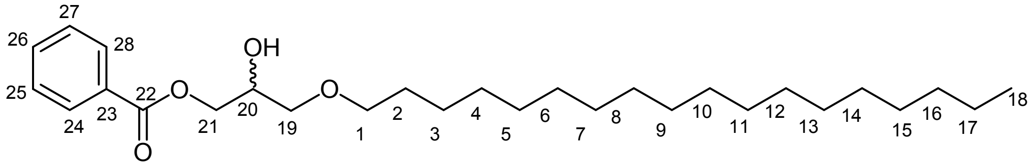

9

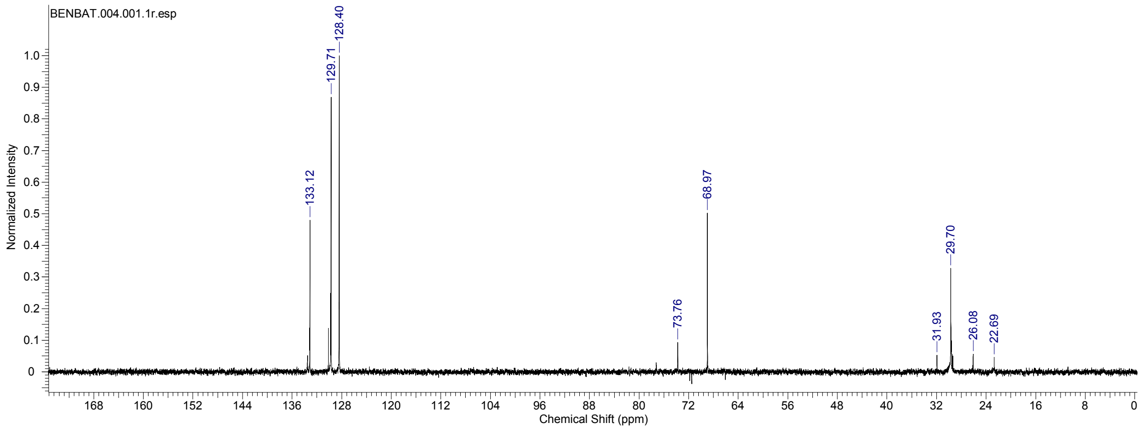

Figure S31. DEPT-90 NMR (100 MHz, $CDCl_3$) spectrum for **9**

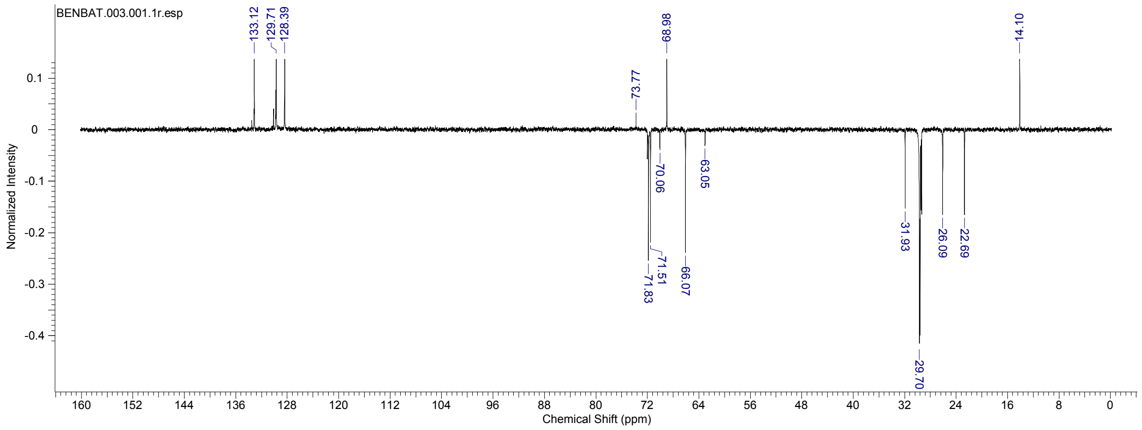

Figure S32. DEPT-135 NMR (100 MHz, $CDCl_3$) spectrum for **9**

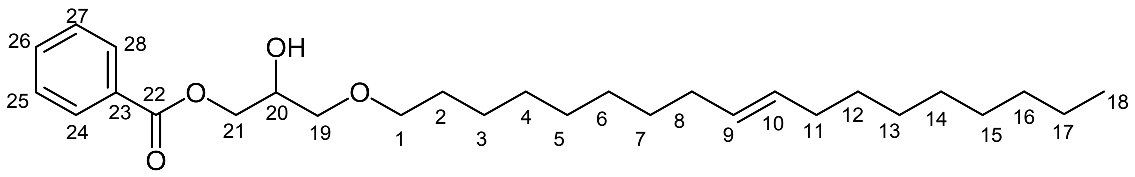

10

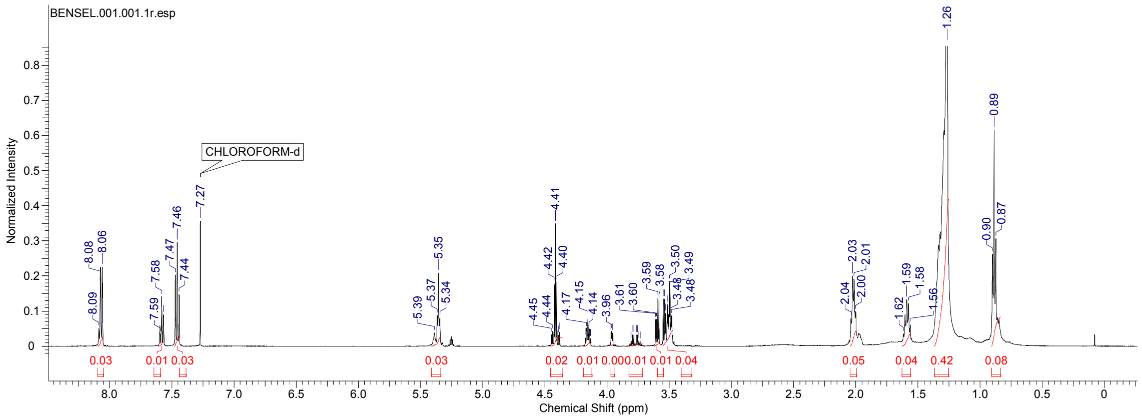

Figure S33. ^{1}H NMR (500 MHz, $CDCl_3$) spectrum for **10**

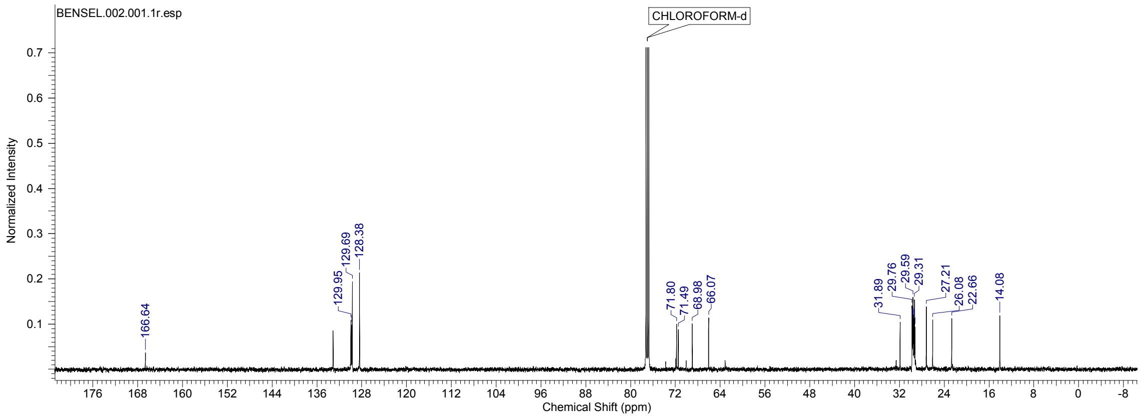

Figure S34. ^{13}C NMR (125 MHz, $CDCl_3$) spectrum for **10**

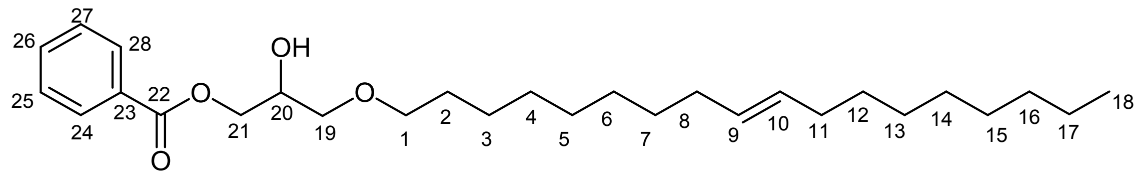

10

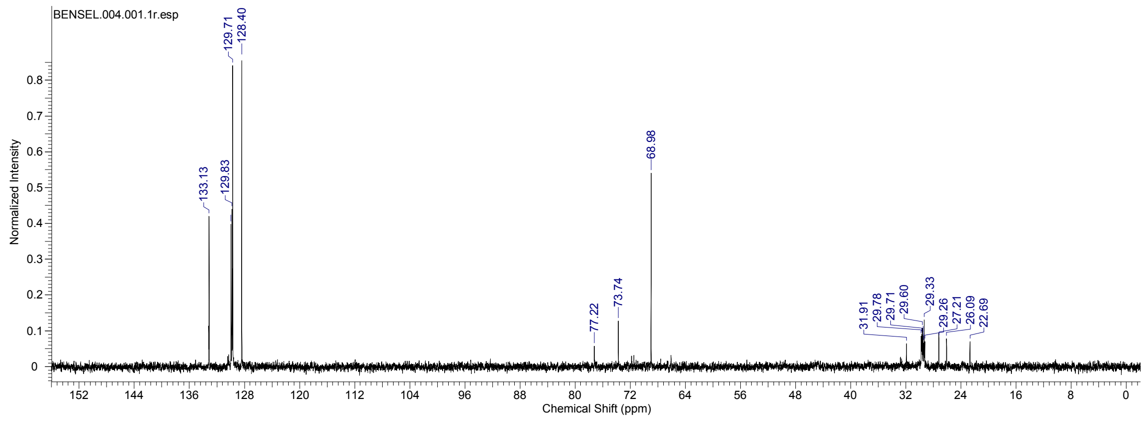

Figure S35. DEPT-90 NMR (125 MHz, $CDCl_3$) spectrum for **10**

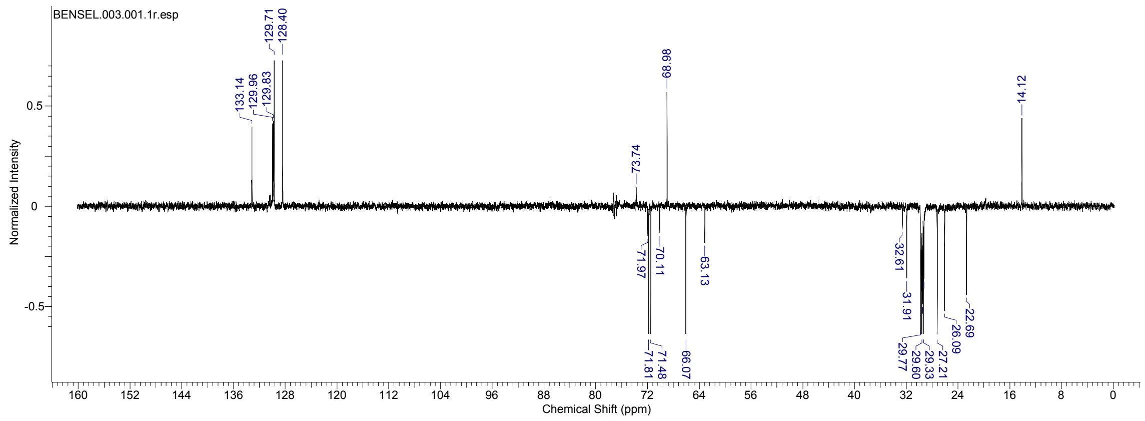

Figure S36. DEPT-135 NMR (125 MHz, $CDCl_3$) spectrum for **10**

6. The 2D NMR (COSY) of 7, 9 and 10

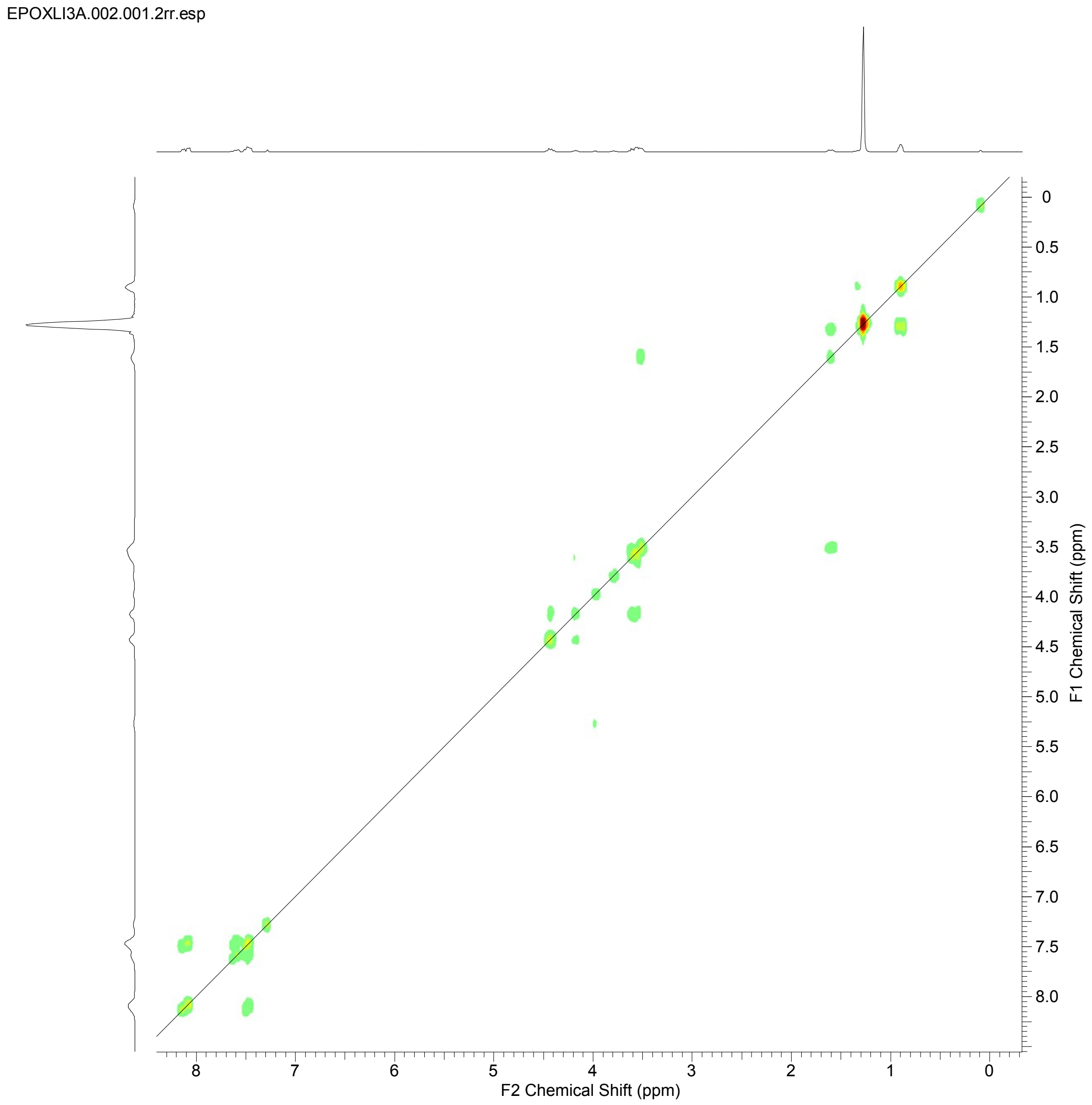

Figure S37. 2D NMR (400 MHz, $CDCl_3$) spectrum for **7**

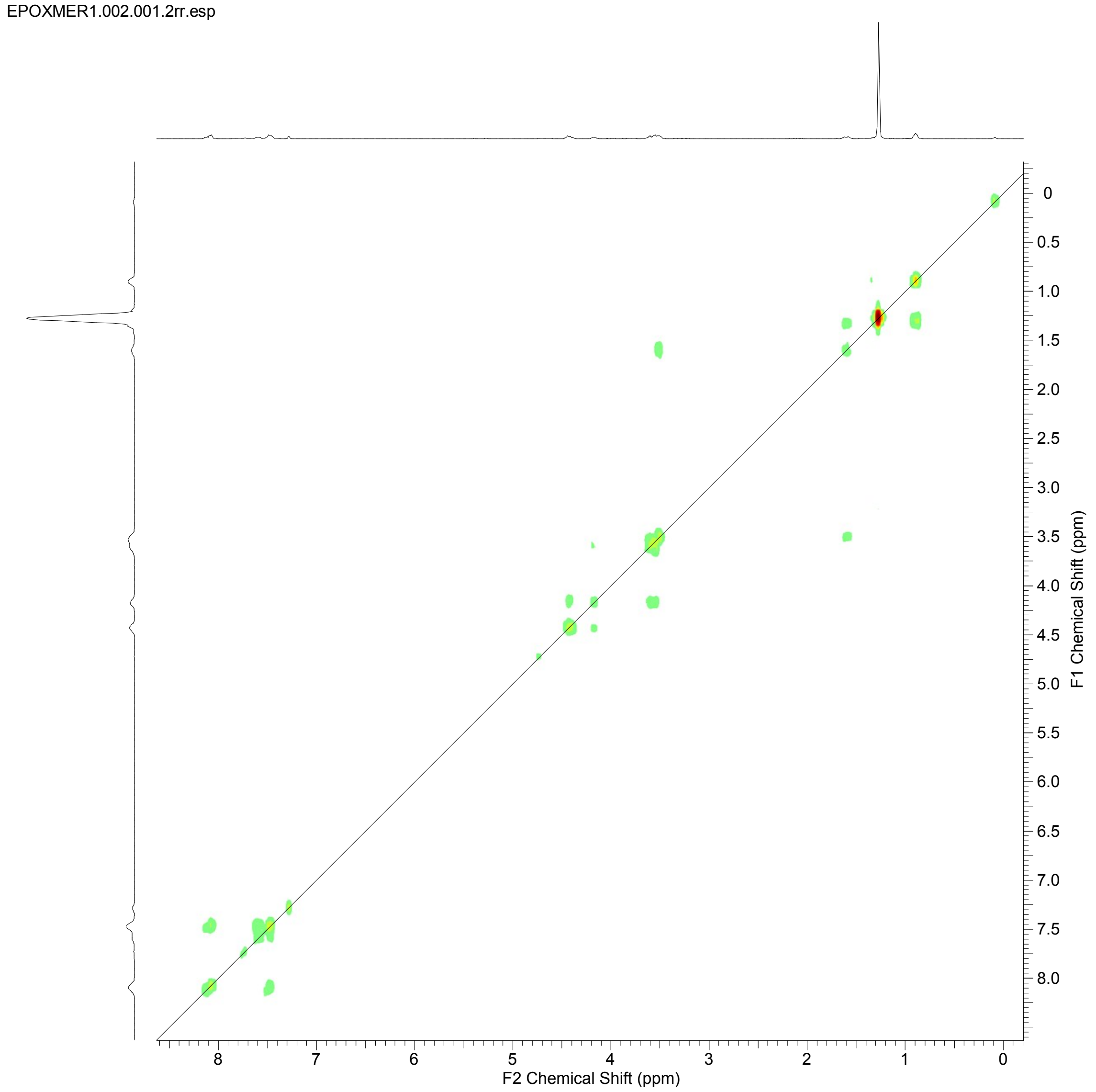

Figure S38. 2D NMR (400 MHz, $CDCl_3$) spectrum for **9**

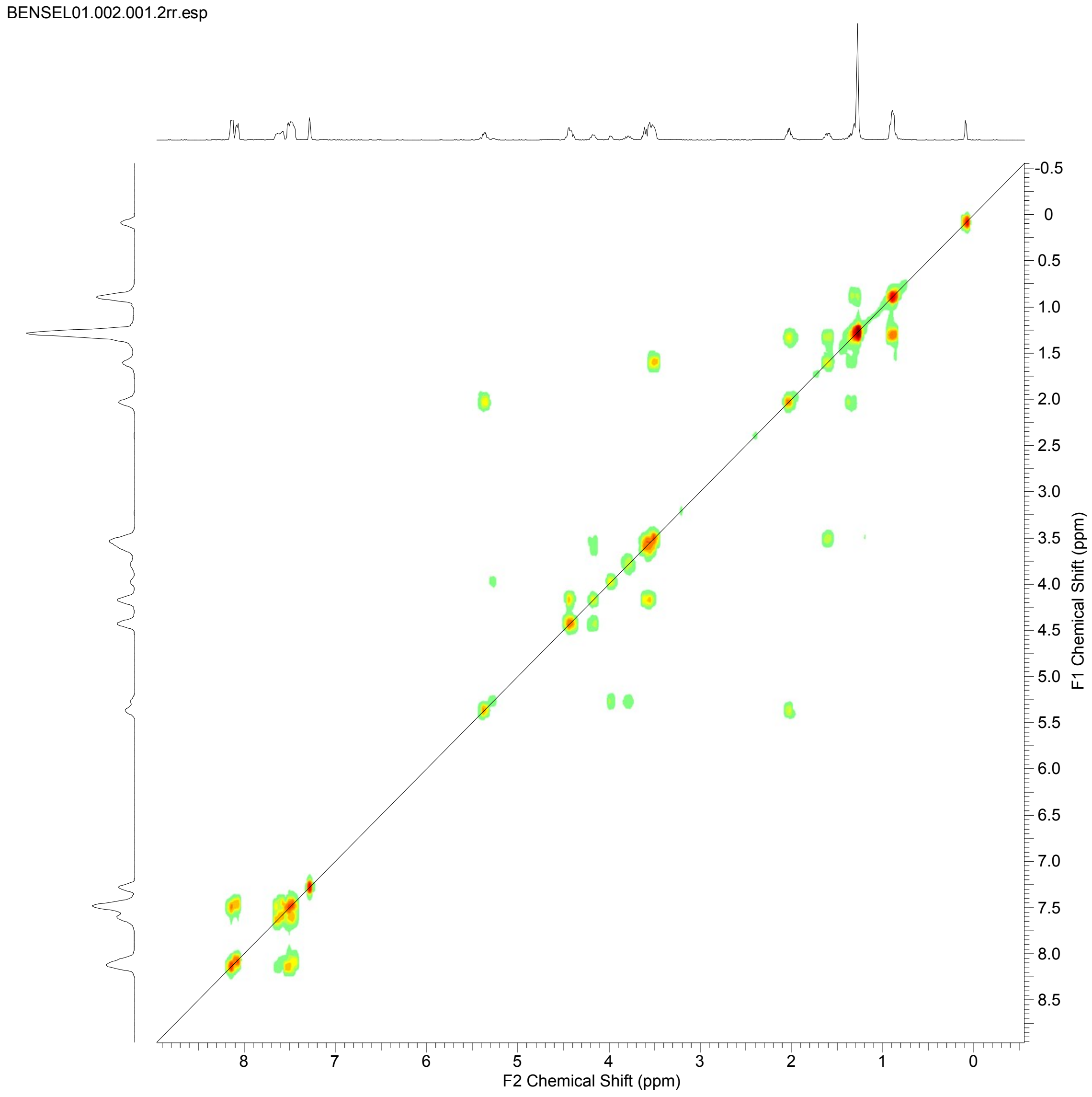

Figure S39. 2D NMR (400 MHz, $CDCl_3$) spectrum for **10**
